# **Long-Term Prognostication for 20 114 Women With Small and Node-Negative Breast Cancer** **(T1abN0)**

**DOI:** 10.1093/jncics/pkaa084

**Published:** 2020-09-26

**Authors:** David Jaraj, Jonas Höijer, Linnea Widman, Johan Ahlgren, Lars-Gunnar Arnesson, Zakaria Einbeigi, Marie Klintman, Eva Vikhe Patil, Malin Sund, Irma Fredriksson, Jonas Bergh, Pettersson Andreas

**Affiliations:** 1 Clinical Epidemiology Division, Department of Medicine, Solna, Karolinska Institutet, Stockholm, Sweden; 2 Department of Surgery, Capio St Görans Hospital, Stockholm, Sweden; 3 Unit of Biostatistics, Institute of Environmental Medicine, Karolinska Institutet, Stockholm, Sweden; 4 Department of Oncology, Faculty of Medicine and Health, Örebro University, Örebro, Sweden; 5 Regional Cancer Centre, Uppsala Örebro Health Care Region, Uppsala, Sweden; 6 Department of Surgery, University Hospital, Linköping, Sweden; 7 Department of Medicine, Southern Älvsborg Hospital, Borås, Sweden; 8 Department of Oncology, Institute of Clinical Sciences, Sahlgrenska Academy, Sahlgrenska University Hospital, Gothenburg, Sweden; 9 Department of Oncology and Radiation Physics, Skåne University Hospital, Lund University, Lund, Sweden; 10 Department of Surgical and Perioperative Sciences/Surgery, Umeå University, Umeå, Sweden; 11 Department of Molecular Medicine and Surgery, Karolinska Institutet, Stockholm, Sweden; 12 Department of Breast, Endocrine and Sarcoma Surgery, Karolinska University Hospital, Stockholm, Sweden; 13 Department of Oncology-Pathology, Karolinska Institutet, Stockholm, Sweden; 14 Breast Cancer Center, Karolinska University Hospital, Stockholm, Sweden

## Abstract

**Background:**

Although small, node-negative breast cancer (ie, T1abN0) constitutes 20% of all newly diagnosed breast cancers, data on prognosis and prognostic factors are limited.

**Methods:**

We conducted a population-based cohort study including 20 114 Swedish women treated for T1abN0 breast cancer from 1977 onward. Patient and tumor data were collected from Swedish breast cancer registries. Cohort subjects were followed through linkage to the Cause of Death Register. We calculated the cumulative incidence of breast cancer–specific and overall death and used Cox regression to estimate hazard ratios (HRs) and 95% confidence intervals (CIs).

**Results:**

During a median follow-up of 9.1 years (range = 0-38), 915 women died of breast cancer and 5416 of any cause. The 10-, 20-, and 30-year cumulative incidences of breast cancer death were 3.4% (95% CI = 3.1% to 3.7%), 7.6% (95% CI = 7.1% to 8.2%), and 10.5% (95% CI = 9.6% to 11.4%), respectively. The multivariable hazard ratios and 95% confidence intervals of breast cancer death were 0.92 (95% CI = 0.88 to 0.97) for each additional calendar year of diagnosis, 4.38 (95% CI = 2.79 to 6.87) for grade 3 vs grade 1 tumors, 0.43 (95% CI = 0.31 to 0.62) for progesterone receptor–positive vs progesterone receptor–negative disease, and 2.01 (95% CI = 0.99 to 4.07) for HER2-positive vs HER2-negative disease. Women with grade 3 vs grade 1 tumors had a 56% increased risk of death from any cause (HR = 1.56, 95% CI = 1.30 to 1.88).

**Conclusions:**

The risk of breast cancer death in T1abN0 disease continues to increase steadily beyond 10 years after diagnosis, has improved over time, and varies substantially by tumor characteristics.

Approximately 20% of breast cancers diagnosed today are 10 mm or smaller and node negative (ie, T1abN0) (1). Whether women with T1abN0 breast cancer should receive adjuvant systemic treatment is controversial. With reported mean 10-year disease-specific survival rates of more than 90% (2-6), the prognosis has historically been considered sufficiently good to exclude adjuvant systemic therapies irrespective of tumor features.

Women with T1abN0 breast cancer have largely been excluded from randomized trials assessing the effect of adjuvant systemic treatment, especially chemotherapy and HER2-targeted therapies. The relative treatment benefits of endocrine therapy and/or chemotherapy are apparent irrespective of tumor size and stage (7-11). However, because data on long-term prognosis and prognostic factors for T1abN0 breast cancer are limited, it remains challenging to select patients for adjuvant treatment. The aim of this study was to assess long-term prognosis and prognostic factors in T1abN0 breast cancer. We conducted a population-based cohort study including 20 114 Swedish women surgically treated for T1abN0 breast cancer from 1977 onward, followed for breast cancer death, all-cause mortality, and metachronous breast cancer.

## Methods

### Study Design and Data Sources

This is a population-based cohort study. Study participants were identified in Sweden’s 6 regional breast cancer registries and the National Breast Cancer Registry and followed for outcomes through linkage to the Cancer Registry, the Total Population Register, and the Cause of Death Register. Linkage was conducted using the national registration number, a unique identifier assigned to all Swedish citizens.

Sweden is divided into 6 healthcare regions [residents in millions (12)]: North (0.9), Uppsala-Örebro (2.1), Stockholm-Gotland (2.4), West (1.9), South-East (1.1), and South (1.9). Before 2000, each region had a collaborative breast cancer group developing regional treatment guidelines. Since 2000, there are national treatment guidelines with regional adjustments. In 1977-1992, each regional group established a regional registry compiling information on patient and tumor characteristics and planned primary treatment of all newly diagnosed breast cancer patients; in the North region, only patients aged younger than 75 years were included in the registry. In 2007, the regional registries were discontinued because the National Breast Cancer Registry was established instead, which includes more than 95% of all newly diagnosed breast cancers patients in Sweden (13).

The Cancer Registry includes all newly diagnosed cancers in Sweden with more than 96% completeness (14). The Total Population Register includes virtually 100% of deaths and 91% of emigrations (15). The Cause of Death Register contains underlying and contributory causes of death with 96% completeness and with a disagreement of 6.9% for breast cancer as the underlying cause of death (16,17).

The study was approved by the Stockholm Regional Ethics Committee (2014/365-31/2), with jurisdiction for all participating sites. The study protocol has been registered at www.clinicaltrials.gov (NCT03390608).

### Study Cohort

**Figure 1.**
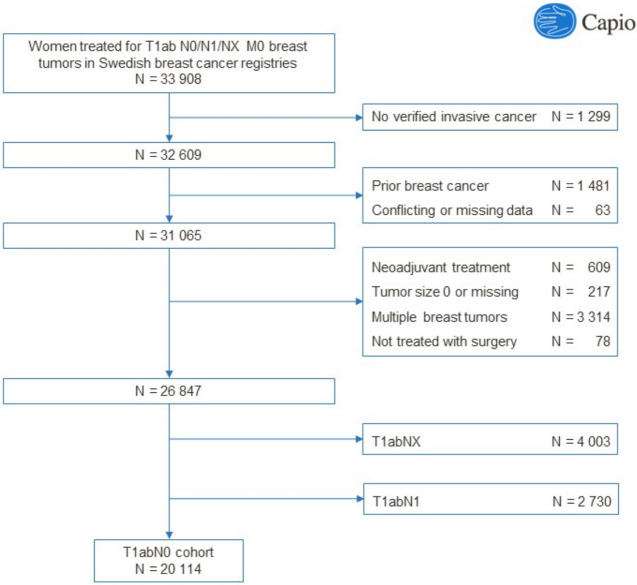
Flow diagram describing the assembly of the study cohort.

A flow diagram describing the assembly of the study cohort is available in Figure 1. Data were retrieved on all women (n = 33 908) in the 6 regional breast cancer registries or the National Breast Cancer Registry who were surgically treated for invasive breast cancer with pathological tumor size 10 mm or smaller, any nodal status (N0, N1, NX), and no metastatic spread (M0). We excluded 1299 women for whom we could not verify a diagnosis of invasive breast cancer (International Classification of Diseases [ICD]-7: 170; ICD-8-9: 174; ICD-10: C50) in the Cancer Registry within an index period of 3 months prior to and 3 months after the date of diagnosis, 1481 women with a breast cancer diagnosis in the Cancer Registry prior to the index period, and 63 women with conflicting data and/or duplicates. We furthermore excluded women receiving neoadjuvant treatment (n = 609), tumor size missing or 0 mm (n = 217), bilateral or multiple breast tumors (n = 3314), treated without surgery (n = 78), unknown nodal status (NX) (n = 4003), or positive nodal status (N1) (n = 2730). The final study cohort included 20 114 women with T1abN0 tumors.

### Exposure, Covariate, and Outcome Data

We retrieved information on the following exposures and covariates from the registries: date of diagnosis (data available for n = 20 114), age at diagnosis (n = 20 114), menopausal status (n = 18 136), screening detection (n = 11 949), tumor size (n = 20 114), tumor grade (n = 12 458), estrogen receptor (ER) status (n = 15 059), progesterone receptor (PR) status (n = 14 757), HER2 status (n = 7930), Ki67 (n = 4007), type of surgery (n = 19 519), adjuvant radiotherapy (n = 15 319), adjuvant endocrine treatment (n = 12 534), and adjuvant chemotherapy (n = 11 533).

The prespecified primary outcome was breast cancer death (BCD), defined as breast cancer (ICD-7: 170; ICD-8-9: 174; ICD-10: C50) listed as the underlying cause of death in the Cause of Death Register (data available to December 31, 2014). Secondary outcomes included death from any cause (data available to July 30, 2016) and metachronous breast cancer (data available to December 31, 2014), defined as ipsilateral or contralateral breast cancer registered in the Cancer Registry at any date after the index period.

### Statistical Analysis

Cohort subjects were followed from the date of diagnosis to the date of BCD, emigration, death from other causes, or end of follow-up (December 31, 2014), whichever occurred first. The cumulative incidence of BCD at different follow-up times was calculated using a competing-risks extension of the Kaplan-Meier estimator (18), with death from other causes considered a competing event. In analyses where any cause of death was considered the outcome, follow-up ended July 30, 2016. Of the 20 114 women in the study cohort, 2 had been diagnosed with breast cancer in January 2015. These 2 women were only included in the survival analysis where all cause death was considered the outcome. Cox proportional hazard regression was used to calculate hazard ratios (HRs) and 95% confidence intervals (CIs). All survival analyses were restricted to women with nonmissing data on relevant exposures and covariates (ie, no imputation was conducted).

“Simple” and “full” regression models were constructed. The simple models were adjusted for year of diagnosis, age at diagnosis, region, and whether the patient was included in the regional breast cancer registries or the National Breast Cancer Registry. The full models were further adjusted for tumor size, tumor grade, and ER status. We created a proxy for the intrinsic subgroups based on tumor grade, ER status, PR status, and HER2 status (luminal A = ER positive [ER+], PR positive [PR+], HER2 negative [HER-], and grade 1-2; luminal B [HER2 negative] = ER positive, HER2 negative, and PR negative and/or grade 3; luminal B [HER2 positive] = ER positive and HER2 positive [HER2+]; HER2 positive [nonluminal] = ER negative [ER-], PR negative [PR-], and HER2 positive; triple negative = ER negative, PR negative, and HER2 negative). Analyses including HER2 status or the intrinsic subgroups were restricted to women diagnosed from January 1, 2005, onward as HER2 status was rarely assessed and registered before then.

We conducted 2 prespecified sensitivity analyses: one where women diagnosed with metachronous breast cancer were censored at the time of diagnosis of the metachronous tumor (sensitivity analysis 1) and one where women with any prior cancer at study entry, except nonmelanoma skin cancer and cancer in situ of the cervix, were excluded (sensitivity analysis 2). The information available in the regional breast cancer registries, including the start date of the registry and the proportion of missing data, differed between regions and over time (Supplementary Tables 1-3, available online), with generally fewer variables and larger proportions of missing data in earlier years. The information available in the National Breast Cancer Registry, in contrast, is homogenous across regions and over time and has a low proportion of missing data (Supplementary Tables 1-3, available online). We therefore conducted 2 additional sensitivity analyses that had not been prespecified in the study protocol; one restricted to women diagnosed from January 1, 2000, onward (sensitivity analysis 3) and one restricted to women in the National Breast Cancer Registry subcohort (sensitivity analysis 4). We also conducted a sensitivity analysis using the Fine and Gray subhazard method to account for competing events (sensitivity analysis 5).

Finally, we conducted the survival analysis using a landmark at 10 years, starting the follow-up at 10 years among women having survived and not being censored at that time. All analyses were conducted using Stata 15.1.

## Results

### Baseline Characteristics

The study cohort included 20 114 women diagnosed with T1abN0 breast cancer between January 14, 1977, and January 20, 2015. The mean age at diagnosis was 60.5  (10.4 ) years. Baseline characteristics are shown in Table 1. Of the tumors, 80.3% were screen-detected, 83.3% T1b (6-10 mm), 88.0% ER positive, 73.0% PR positive, 43.5% grade 1, 43.4% grade 2, 13.1% grade 3, and 9.1% HER2 positive. Of the women, 77.3% underwent partial mastectomy, 81.7% received adjuvant radiotherapy (91.0% after partial mastectomy and 15.3% after mastectomy), 53.9% adjuvant endocrine therapy (60.9% of ER-positive cases), and 7.1% adjuvant chemotherapy. Treatment patterns varied by tumor characteristics with generally more extensive treatment for more aggressive tumors (Supplementary Tables 4 and 5, available online).

**Table 1.** Baseline characteristics in 20 114 women diagnosed with T1abN0 breast cancer 1977 to 2015

Characteristic	No. (%)
Total	20 114 (100)
Age at diagnosis, y	
<35	172 (0.9)
35-44	1239 (6.2)
45-54	4303 (21.4)
55-64	6586 (32.7)
65-74	6585 (32.7)
≥75	1229 (6.1)
Menopausal status^a^^,b^	
Premenopausal	3047 (16.8)
Postmenopausal	15 089 (83.2)
Unknown	1978
Screening detected^a^^,c^	
Yes	9591 (80.3)
No	2358 (19.7)
Unknown	8165
Tumor size	
≤5 mm (T1a)	3367 (16.7)
6-10 mm (T1b)	16 747 (83.3)
Tumor grade^a^	
1	5416 (43.5)
2	5411 (43.4)
3	1631 (13.1)
Unknown	7656
ER status^a^	
Positive	13 255 (88.0)
Negative	1804 (12.0)
Unknown	5055
PR status^a^	
Positive	10 777 (73.0)
Negative	3980 (27.0)
Unknown	5357
HER2 status^a^	
Positive	721 (9.1)
Negative	7209 (90.9)
Unknown	12 184
Proliferation^a^	
Low, ≤20%	2972 (74.2)
High, >20%	1035 (25.8)
Unknown	16 107
Intrinsic subgroups^a^^,d^	
Luminal A	5300 (68.7)
Luminal B, HER2 negative	1283 (16.6)
Luminal B, HER2 positive	498 (6.5)
HER2 positive, nonluminal	205 (2.7)
Triple negative	432 (5.6)
Unknown	12 396
Type of surgery^a^	
Partial mastectomy	15 079 (77.3)
Mastectomy	4440 (22.7)
Unknown	595
Adjuvant radiotherapy^a^	
No	2810 (18.3)
Yes	12 509 (81.7)
Unknown	4795
Adjuvant endocrine therapy^a^	
No	5781 (46.1)
Yes	6753 (53.9)
Unknown	7580
Adjuvant chemotherapy^a^	
No	10 709 (92.9)
Yes	824 (7.1)
Unknown	8581
Adjuvant trastuzumab^a^	
Yes	329 (NA)
Unknown	19 785

a Percentages are calculated without including women in the “unknown” category in the denominator. ER = estrogen receptor; PR = progesterone receptor.

b Women with missing registry data on menopausal status aged younger than 45 years were considered premenopausal and those aged 55 years or older as postmenopausal.

c Data on whether the tumor was screening detected or clinically detected is restricted to women aged 40-74 years.

d Luminal A = ER positive, PR positive, HER2 negative, and grade 1-2; Luminal B (HER2 negative) = ER positive, HER2 negative, and PR negative, and/or grade 3; Luminal B (HER2 positive) = ER positive, and HER2 positive; HER2 positive (nonluminal) = ER negative, PR-negative, and HER2 positive; triple negative = ER negative, PR negative, and HER2 negative.

### Cumulative Incidences and Hazard Ratios of BCD

**Figure 2.**
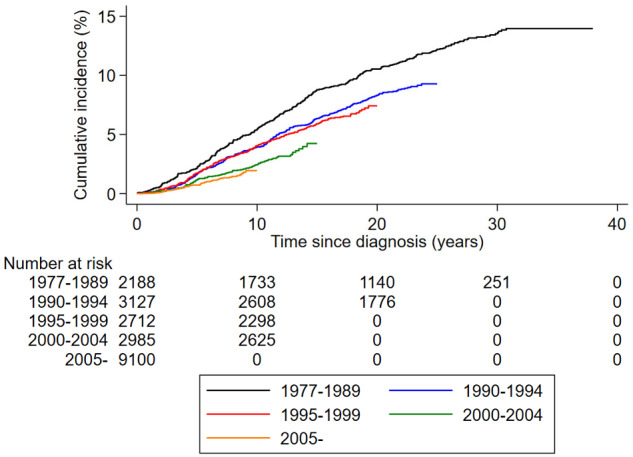
Cumulative incidence of breast cancer death in women with T1abN0 breast cancer stratified by year of diagnosis.

**Figure 3.**
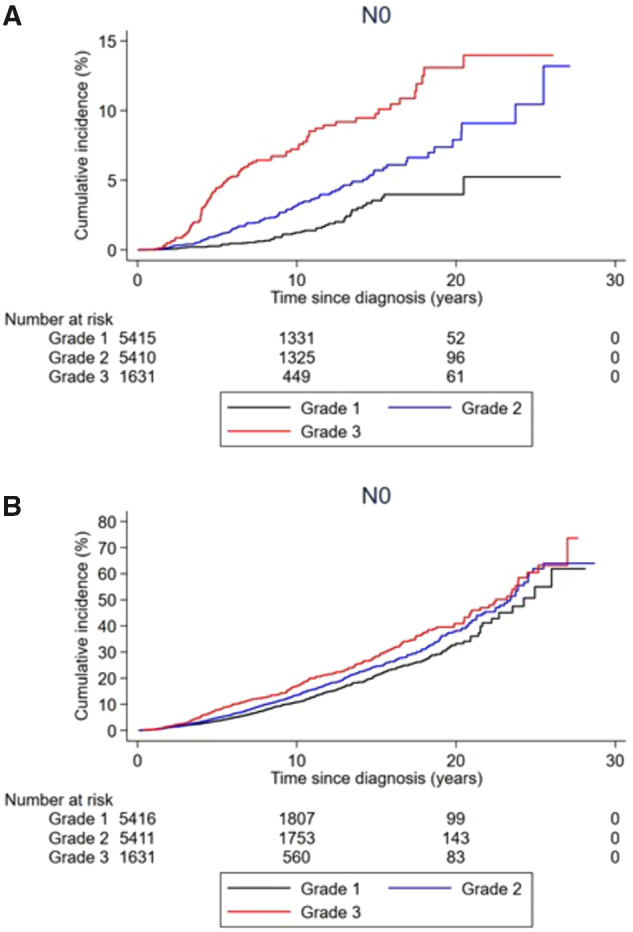
Cumulative incidence of breast cancer death and death from any cause in women with T1abN0 breast cancer stratified by tumor grade. **A**) Cumulative incidence of breast cancer death and **B**) cumulative incidence of death by any cause are shown.

**Figure 4.**
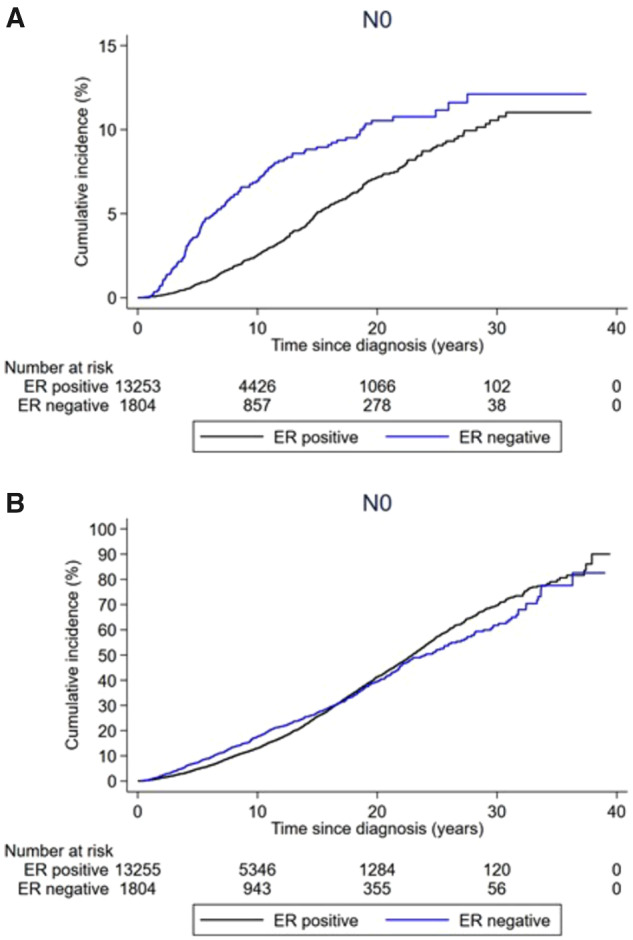
Cumulative incidence of death from breast cancer and death from any cause among women with T1abN0 breast cancer stratified by ER status. **A**) Cumulative incidence of breast cancer death and **B**) cumulative incidence of death by any cause are shown.

Table 2 and Figures 2-4 shows cumulative incidences of BCD overall and by patient and tumor characteristics. During a median follow-up of 9.1 years (range = 0-38 years), 915 women died of breast cancer. The cumulative incidence of BCD at 10, 20, and 30 years of follow-up was 3.4% (95% CI = 3.1% to 3.7%), 7.6% (95% CI = 7.1% to 8.2%), and 10.5% (95% CI = 9.6% to 11.4%), respectively. The 10-year cumulative incidence decreased from 5.5% (95% CI = 4.6% to 6.5%) among those diagnosed between 1977 and 1989 to 2.5% (95% CI = 1.9% to 3.1%) among those diagnosed between 2000 and 2004. It also varied by patient and tumor characteristics, especially by age and tumor grade: 11.2% (95% CI = 6.5% to 17.2%) among those aged younger than 35 years, 3.2% (95% CI = 2.7% to 3.9%) among those aged 45-54 years, and 5.1% (95% CI = 3.8% to 6.7%) among those aged 75 years and older. For tumor grade, it varied from 1.2% (95% CI = 0.9% to 1.8%) for grade 1 to 7.4% (95% CI = 5.9% to 9.2%) for grade 3. ER-positive vs ER-negative tumors had a lower cumulative incidence of BCD, but the difference diminished over time (Table 2 and Figure 4).

**Table 2.** Cumulative incidences and hazard ratios of death from breast cancer by patient and tumor characteristics in 20 112 women with T1abN0 breast cancer

Characteristic	Cumulative incidence % (95% CI)			Hazard ratio (95% CI)	
	10 years	20 years	30 years	Simple model^a^	Full model^b^
All	3.4 (3.1 to 3.7)	7.6 (7.1 to 8.2)	10.5 (9.6 to 11.4)	NA	NA
Year of diagnosis, events/No.	**—**	**—**	**—**	915/20 112	190/11 188
1977-1989	5.5 (4.6 to 6.5)	10.6 (9.3 to 11.9)	13.6 (12.2 to 15.2)	Referent	Referent
1990-1994	3.9 (3.3 to 4.7)	8.3 (7.4 to 9.3)	—	0.72 (0.60 to 0.86)	0.74 (0.19 to 2.87)
1995-1999	4.0 (3.3 to 4.8)	—	—	0.64 (0.52 to 0.78)	0.61 (0.16 to 2.31)
2000-2004	2.5 (1.9 to 3.1)	—	—	0.44 (0.34 to 0.56)	0.35 (0.09 to 1.34)
2005-end of follow up	—	—	—	0.36 (0.25 to 0.51)	0.29 (0.07 to 1.14)
Age at diagnosis, y, events/No.	—	—	—	915/20 112	190/11 188
<35	11.2 (6.5 to 17.2)	26.7 (17.9 to 36.3)	—	3.11 (2.10 to 4.60)	1.56 (0.56 to 4.36)
35-44	5.3 (3.9 to 6.9)	11.6 (9.3 to 14.2)	18.6 (14.1 to 23.5)	1.50 (1.18 to 1.92)	1.39 (0.80 to 2.39)
45-54	3.2 (2.7 to 3.9)	7.1 (6.1 to 8.2)	—	Referent	Referent
55-64	3.0 (2.5 to 3.5)	7.7 (6.7 to 8.7)	10.4 (8.9 to 12.1)	1.15 (0.96 to 1.38)	0.94 (0.64 to 1.39)
65-74	3.1 (2.6 to 3.6)	7.0 (6.1 to 7.9)	7.9 (6.9 to 9.1)	1.23 (1.02 to 1.49)	1.05 (0.70 to 1.57)
≥75	5.1 (3.8 to 6.7)	—	—	2.10 (1.55 to 2.85)	1.67 (0.83 to 3.34)
Menopausal status, events/No.	—	—	—	822/18 134	166/10 126
Premenopausal	4.3 (3.5 to 5.2)	10.5 (9.0 to 12.1)	16.7 (13.8 to 19.9)	Referent	Referent
Postmenopausal	3.2 (2.9 to 3.6)	7.2 (6.6 to 7.8)	9.0 (8.1 to 9.9)	0.89 (0.63 to 1.25)	1.55 (0.75 to 3.19)
Screening detected, events/No.	—	—	—	327/11 948	121/8813
No	3.0 (2.2 to 4.0)	8.1 (6.3 to 10.1)	—	Referent	Referent
Yes	2.7 (2.3 to 3.2)	6.9 (5.9 to 7.9)	—	0.88 (0.68 to 1.15)	1.19 (0.76 to 1.88)
Tumor size, events/No.	—	—	—	915/20 112	190/11 188
≤5 mm	3.6 (2.9 to 4.4)	6.9 (5.7 to 8.2)	—	Referent	Referent
6 to ≤10 mm	3.4 (3.0 to 3.7)	7.8 (7.2 to 8.4)	10.8 (9.9 to 11.9)	1.07 (0.89 to 1.28)	0.73 (0.50 to 1.05)
Tumor grade, events/No.	—	—	—	275/12 456	190/11 188
1	1.2 (0.9 to 1.8)	5.2 (2.9 to 8.5)	—	Referent	Referent
2	3.2 (2.6 to 3.9)	7.9 (6.0 to 10.2)	—	2.22 (1.61 to 3.07)	2.44 (1.65 to 3.60)
3	7.4 (5.9 to 9.2)	14.0 (10.6 to 17.8)	—	4.55 (3.23 to 6.41)	4.38 (2.79 to 6.87)
ER status, events/No.	—	—	—	516/15 057	190/11 188
Negative	7.0 (5.7 to 8.4)	10.5 (8.8 to 12.5)	—	Referent	Referent
Positive	2.5 (2.2 to 2.9)	7.2 (6.4 to 8.0)	10.8 (9.3 to 12.4)	0.65 (0.53 to 0.80)	0.69 (0.48 to 0.99)
PR status, events/No.	—	—	—	478/14 755	181/11 051
Negative	5.3 (4.5 to 6.2)	9.8 (8.5 to 11.2)	12.7 (10.7 to 14.8)	Referent	Referent
Positive	2.0 (1.7 to 2.4)	6.3 (5.5 to 7.3)	—	0.55 (0.46 to 0.66)	0.43 (0.31 to 0.62)
HER2 status, events/No.^c^	5 y	NA	NA	45/7477	44/7332
Negative	0.6 (0.4 to 0.8)	—	—	Referent	Referent
Positive	2.7 (1.4 to 4.7)	—	—	3.87 (2.01 to 7.45)	2.01 (0.99 to 4.07)
Intrinsic subgroups, events/No.^c^	5 y	NA	NA	43/7332	43/7286
Luminal A	0.2 (0.1 to 0.5)	—	—	Referent	Referent
Luminal B, HER2 negative	1.3 (0.6 to 2.4)	—	—	3.71 (1.60 to 8.60)	2.87 (1.16 to 7.09)
Luminal B, HER2 positive	2.1 (0.9 to 4.3)	—	—	7.27 (2.90 to 18.2)	5.03 (1.92 to 13.2)
HER2 positive, nonluminal	—	—	—	10.7 (3.68 to 31.0)	5.76 (1.76 to 18.9)
Triple negative	3.4 (1.5 to 6.4)	—	—	8.20 (3.28 to 20.5)	4.89 (1.76 to 13.6)

a The simple model is adjusted for year of diagnosis (continuous), age at diagnosis (categorical: <35, 35-44, 45-54, 55-64, 65-74, ≥75), region (categorical: Stockholm-Gotland, Uppsala-Örebro, South-East, South, West, North), and registry source (categorical: regional breast cancer registries, the National Breast Cancer Registry). “—” indicates data not available for analysis. CI = confidence interval; ER = estrogen receptor; PR = progesterone receptor.

b The full model is adjusted for the same variables as the simple model plus tumor size (categorical: T1a, T1b), tumor grade (categorical: 1, 2, 3), and ER status (categorical: positive, negative).

c Analyses of HER2 status and the intrinsic subgroups are restricted to women diagnosed January 1, 2005, onward.

Table 2 shows hazard ratios for BCD for the simple and full regression models. In the simple models, year of diagnosis (2005- to end of follow up vs 1977-1989: HR = 0.36, 95% CI = 0.25 to 0.51), age at diagnosis (younger than 35 years vs 45-54 years: HR = 3.11, 95% CI = 2.10 to 4.60; 75 years or older vs 45-54 years: HR = 2.10, 95% CI = 1.55 to 2.85), tumor grade (3 vs 1: HR = 4.55, 95% CI = 3.23 to 6.41), ER status (ER+ vs ER-: HR = 0.65, 95% CI = 0.53 to 0.80), PR status (PR+ vs PR-: HR = 0.55, 95% CI = 0.46 to 0.66), and HER2 status (HER+ vs HER-: HR = 3.87, 95% CI = 2.01 to 7.45) were associated with risk of BCD. Although the precision was lower in the fully adjusted models, the associations remained largely unchanged except for age at diagnosis (younger than 35 years vs 45-54 years: HR = 1.56, 95% CI = 0.56 to 4.36; 75 years or older vs 45-54 years: HR = 1.67, 95% CI = 0.83 to 3.34), and HER2-status (HER2+ vs HER2-: HR = 2.01, 95% CI = 0.99 to 4.07). The hazard ratios from the fully adjusted models were 0.92 (95% CI = 0.88 to 0.97) for each additional calendar year of diagnosis, 4.38 (95% CI = 2.79 to 6.87) for grade 3 vs grade 1 tumors, and 0.43 (95% CI = 0.31 to 0.62) for PR-positive vs PR-negative disease. In analyses including the intrinsic subgroups, compared with women with luminal A tumors, women with triple-negative tumors (multivariable HR = 4.89, 95% CI = 1.76 to 13.6), HER2-positive (nonluminal) tumors (multivariable HR = 5.76, 95% CI = 1.76 to 18.9), and luminal B (HER2-positive) tumors (HR = 5.03, 95% CI = 1.92 to 13.2) had the highest and similar relative risks of BCD.

The associations were generally similar in the 5 sensitivity analyses (Supplementary Tables 6-10, available online). Results from the analysis starting follow-up at 10 years after diagnosis are shown in Supplementary Table 11 (available online). The major differences compared with the results from the main analysis were that the association between tumor grade and BCD was attenuated (3 vs 1: HR = 1.65, 95% CI = 0.87 to 3.11 in the simple model and HR = 2.60, 95% CI = 1.02 to 6.61 in the full model) and that ER positivity vs ER negativity was associated with a higher, not lower, risk of BCD (ER+ vs ER-: HR = 1.55, 95% CI = 1.06 to 2.27 in the simple model and HR = 1.96, 95% CI = 0.79 to 4.86 in the full model).

### Cumulative Incidences and Hazard Ratios of Death From Any Cause and Metachronous Breast Cancer

Supplementary Table 12 (available online) shows cumulative incidences and hazard ratios for death from any cause. During follow-up, 5416 women died of any cause. In the full regression models, old age at diagnosis (75 years or older vs 45-54 years: HR = 10.9, 95% CI = 8.61 to 13.7), being postmenopausal vs premenopausal at diagnosis (HR = 1.79, 95% CI = 1.14 to 2.82), and higher tumor grade (3 vs 1: HR = 1.56, 95% CI = 1.30 to 1.88) were associated with a higher risk of death from any cause (Figure 3). The pattern of better shorter-term but similar longer-term prognosis for ER-positive vs ER-negative tumors was evident also when death from any cause was the outcome (Figure 4). None of the patient or tumor characteristics were associated with risk of metachronous breast cancer (Supplementary Table 13, available online).

## Discussion

The 10-year cumulative incidence of BCD was 3.4% in our cohort, in agreement with prior reports including a 4.0% estimate from Surveillance, Epidemiology, and End Results (19,20). For T1abN0 tumors, data on prognosis beyond 10 years are limited. A few publications have reported 20-year disease-free survival estimates of 70%-88% (20-22). In clinical decision-making, data on long-term prognosis in women not receiving adjuvant treatment are especially important. A Finnish study of 80 women diagnosed with T1abN0 tumors between 1945 and 1976 not receiving adjuvant systemic treatment reported 10- and 20-year BCD rates of 6% and 8%, respectively (4). A recently published Swedish study reported a 15-year breast cancer–specific survival rate of 93.7% among 1543 patients diagnosed between 1997 and 2002 with T1abN0 grade 1-2 tumors of whom 12% received adjuvant endocrine therapy and 0% adjuvant chemotherapy (5). Based on our data, the 10-year absolute risk of BCD among women not receiving adjuvant systemic treatment is around 6%; the observed 10-year cumulative incidence of BCD among those diagnosed between 1977 and 1989, a period during which adjuvant systemic treatment was recommended only in selected cases, was 5.5%. However, given the substantially improved prognosis over time, it is unclear whether this observed 10-year cumulative incidence of BCD is applicable for contemporary women. Our data also indicate that the risk of BCD continues to increase steadily beyond 10 years after diagnosis. This finding is corroborated by a recent Early Breast Cancer Trialists’ Collaborative Group meta-analysis assessing 20-year prognosis among women with ER-positive tumors treated with 5 years of adjuvant endocrine treatment (10). In the meta-analysis, including only 5527 women, the 20-year risk of BCD in the T1N0 subgroup (ie, T1abcN0) was 12%, and in analyses starting follow-up at 5 years postdiagnosis, the risk of BCD after 15 years was 5% for T1abN0 and 8% for T1cN0 tumors. To the best of our knowledge, before our study no such long-term follow-up data existed for ER-negative T1abN0 tumors.

Our data suggest that standard prognostic factors in larger or node-positive breast cancer retain prognostic relevance in the T1abN0 subgroup. ER negativity, PR negativity, HER2 positivity, and higher tumor grade were associated with poorer prognosis, in agreement with results from most prior smaller studies (3,4,19,23-27). Likewise, the 5-year cumulative incidence of BCD was 0.2% for luminal A tumors vs 3.4% for triple-negative tumors. Tumor grade in particular was a strong prognostic factor in our study. Women with grade 3 vs grade 1 tumors were more than 400% more likely to die from breast cancer and more than 50% more likely to die from any cause. Interestingly, the association between tumor grade and BCD was lower in the analysis restricted to women being alive 10 year after the diagnosis, suggesting that the prognostic utility of tumor grade diminishes over time.

In our study, women with ER-positive vs ER-negative tumors had a better 10-year prognosis but more similar longer-term prognosis, consistent with prior studies showing that late relapses are common in women with larger or node-positive ER-positive tumors (28). These results add further to the notion that T1abN0 tumors behave biologically as larger tumors. The high cumulative incidence of BCD among younger women is notable and in line with previous findings for both T1abN0 and larger tumors (20,24,29). We observed no difference in prognosis comparing T1aN0 with T1bN0, which is probably explained by a larger proportion of women with T1bN0 receiving active treatment, rather than tumor size not being a prognostic factor in the T1abN0 subgroup. We observed successively improved prognosis over time. The 10-year cumulative incidence of BCD was, for example, 2.5% among those diagnosed from 2000 to 2004 vs 5.5% among those diagnosed from 1977 to 1989. Several explanations are possible for this finding, including more and better adjuvant treatment over time. The improved prognosis over time can also be explained by other factors, such as earlier detection and active treatment of aggressive T1abN0 tumors, or by increasing overdiagnosis of indolent T1abN0 tumors because of mammography screening. The latter explanation seems unlikely, however, as we observed no appreciable difference in prognosis for tumors being screening detected vs not screening detected. Although we are unable to pinpoint the precise mechanisms, our data suggest that the changes over time in breast cancer screening, diagnosis, and treatment have led to improved prognosis not only for women with larger or node-positive tumors but also for women with T1abN0 breast cancer. Key strengths of this study include its population-based design, large sample size, long-term follow-up, and the main outcome being BCD. Key limitations of these real-world data when assessing prognosis and prognostic factors are missing data and misclassification. We addressed the missing data issue by conducting sensitivity analyses restricted to women diagnosed from 2000 onward and, respectively, to women in the National Breast Cancer Registry subcohort, which yielded largely similar or even more pronounced relative risks. Registry data are prone to misclassification, and we conducted no medical chart review or re-review of pathological material. However, we have no reason to suspect that the key findings of this study are due to misclassification. Indeed, any bias stemming from misclassification should primarily be at random and drive the associations toward the null. We did not focus this study on assessing treatment effects, which in these types of studies are prone to bias because of confounding by indication, selection bias, and lack of data on all relevant treatment selection factors. It is important to note, however, that the relative treatment benefits of endocrine therapy and/or chemotherapy in randomized trials are independent of tumor size and stage (7-11), suggesting that the relative treatment benefits are similar also among women with T1abN0 tumors.

In conclusion, the data from this study suggest that the risk of BCD among women with T1abN0 breast cancer increases steadily with time since diagnosis, has improved over time, and varies substantially by tumor characteristics. For certain subgroups, for example, women with grade 3 tumors, the risk of BCD was considerable. The premise that prognosis for all women with T1abN0 breast cancer is sufficiently good to exclude adjuvant treatment irrespective of tumor features should be abandoned. Women with T1abN0 breast cancer should be considered for adjuvant therapy interventions, in particular those with aggressive tumor features or long life expectancy. Randomized clinical trials including patients with T1abN0 breast cancer are warranted.

## Funding

This work was supported by grants from the Swedish Breast Cancer Association and the Swedish Society of Medicine (SLS-502451).

## Notes

**The role of the funder:** The funders had no role in the design of the study; the collection, analysis, and interpretation of the data; the writing of the manuscript; and the decision to submit the manuscript for publication.

**Disclosures:** The authors declare no potential conflicts of interest.

**Author contributions:** Conceptualization, DJ, JA, LGA, ZE, MK, EVP, MS, IF, JB, and AP; Formal analysis, JH and LW; Investigation, DJ, JA, LGA, ZE, MK, EVP, MS, IF, JB, and AP; Resources, AP; Data curation, DJ, JH, LW, and AP; Writing - original draft, DJ; Writing - review & editing, all authors; Supervision, IF, JB, and AP; Project administration, AP; Funding acquisition, IF, JB, and AP.

**Acknowledgments:** We are very grateful to the data managers Ulla Johansson; Marit Holmqvist; Chenyang Zhang; Elisabeth Johansson; Helena Fohlin, PhD; Barbro Numan Hellquist; and David Norman at the regional breast cancer registries and the National Breast Cancer Registry and to Dr Per Malmström at the Division of Oncology and Pathology, Department of Clinical Sciences, Lund University, Lund, Sweden, for his valuable insights and contributions.

## Data Availability

The data underlying this article cannot be shared publicly for privacy of individuals who participated in the study.

## Supplementary Material

pkaa084_Supplementary_Data
