# Supplementary material for: Long-Term Prognostication for 20 114 Women With Small and Node-Negative Breast Cancer (T1abN0)
Source: JNCI Cancer Spectr. 2020 Sep 26;5(1):pkaa084. doi: 10.1093/jncics/pkaa084 (PMC7791632; doi:10.1093/jncics/pkaa084)
Supplement: pkaa084_Supplementary_Data [file pkaa084_supplementary_data.pdf]

Supplementary Table 1. Number of patients with T1abN0 breast cancer per region and year of diagnosis.							
**Year of diagnosis**	**Stockholm-Gotland**	**Uppsala-Örebro**	**South-East**	**South**	**West**	**North**	**Total No.**
1977	64	NA	NA	NA	NA	NA	64
1978	64	NA	NA	NA	NA	NA	64
1979	69	NA	NA	NA	NA	NA	69
1980	63	NA	NA	NA	NA	NA	63
1981	90	NA	NA	NA	NA	NA	90
1982	117	NA	NA	40	NA	NA	157
1983	96	NA	19	35	NA	NA	150
1984	89	NA	41	43	NA	NA	173
1985	90	NA	33	56	NA	NA	179
1986	111	NA	43	59	NA	NA	213
1987	101	NA	89	48	NA	5	243
1988	120	NA	97	89	NA	25	331
1989	118	NA	85	84	81	24	392
1990	145	NA	76	121	139	74	555
1991	192	NA	81	123	153	57	606
1992	134	79	96	106	101	75	591
1993	129	174	74	108	108	63	656
1994	175	165	69	116	121	73	719
1995	145	204	74	106	113	67	709
1996	155	159	69	102	108	68	661
1997	157	72	54	58	97	44	482
1998	111	69	45	52	99	54	430
1999	101	95	52	50	83	49	430
2000	97	80	46	46	104	29	402
2001	97	96	41	58	85	21	398
2002	133	110	71	83	87	56	540
2003	180	201	107	138	146	76	848

2004	196	178	86	129	124	84	797
2005	168	160	101	132	123	77	761
2006	181	209	73	147	128	70	808
2007	194	181	95	179	128	76	853
2008	195	165	96	158	153	104	871
2009	179	181	90	131	125	77	783
2010	213	203	83	205	138	108	950
2011	198	202	97	204	160	94	955
2012	213	230	70	207	171	87	978
2013	239	226	103	201	204	94	1,067
2014	255	228	117	198	192	84	1,074
2015	0	1	0	1	0	0	2
Total N	5,374	3,668	2,373	3,613	3,271	1,815	20,114

Supplementary Table 2. Baseline characteristics among 20,114 women with T1abN0 breast cancer overall and stratified by registry source.			
**Characteristic**	**Total No. (%)**	**Regional registries No. (%)**	**National registry No. (%)**
Total, No.	20,114	13,248	6,866
Year of diagnosis, min-max	1977-2015	1977-2007	2007-2015
Age at diagnosis, y			
<35	172 (0.9)	132 (1.0)	40 (0.6)
35-44	1,239 (6.2)	864 (6.5)	375 (5.5)
45-54	4,303 (21.4)	3,109 (23.5)	1,194 (17.4)
55-64	6,586 (32.7)	4,436 (33.5)	2,150 (31.3)
65-74	6,585 (32.7)	3,909 (29.5)	2,676 (39.0)
≥75	1,229 (6.1)	798 (6.0)	431 (6.3)
Menopausal status*†			
Premenopausal	3,047 (16.8)	1,981 (16.9)	1,066 (16.6)
Postmenopausal	15,089 (83.2)	9,732 (83.1)	5,357 (83.4)
Unknown	1,978	1,535	443
Screening detected*‡			
Yes	9,591 (80.3)	4,432 (78.4)	5,159 (81.9)
No	2,358 (19.7)	1,219 (21.6)	1,139 (18.1)
Unknown	8,165	7,597	568
Tumor size			
≤5 mm	3,367 (16.7)	2,145 (16.2)	1,222 (17.8)
6-10 mm	16,747 (83.3)	11,103 (83.8)	5,644 (82.2)
Tumor grade*			
I	5,416 (43.5)	2,463 (43.1)	2,953 (43.8)
II	5,411 (43.4)	2,451 (42.9)	2,960 (43.9)
III	1,631 (13.1)	804 (14.1)	827 (12.3)
Unknown	7,656	7,530	126

ER status*			
Positive	13,255 (88.0)	7,042 (84.9)	6,213 (91.8)
Negative	1,804 (12.0)	1,252 (15.1)	552 (8.2)
Unknown	5,055	4,954	101
PR status*			
Positive	10,777 (73.0)	5,475 (68.5)	5,302 (78.4)
Negative	3,980 (27.0)	2,522 (31.5)	1,458 (21.6)
Unknown	5,357	5,251	106
HER2 status*			
Positive	721 (9.1)	166 (11.1)	555 (8.6)
Negative	7,209 (90.9)	1,334 (88.9)	5,875 (91.4)
Unknown	12,184	11,748	436
Proliferation*			
Low (≤20%)	2,972 (74.2)	632 (73.6)	2,340 (74.3)
High (>20%)	1,035 (25.8)	227 (26.4)	808 (25.7)
Unknown	16,107	12,389	3,718
Intrinsic subgroups*§			
Luminal A	5,300 (68.7)	843 (61.0)	4,457 (70.3)
Luminal B (HER2-negative)	1,283 (16.6)	280 (20.3)	1,003 (15.8)
Luminal B (HER2-positive)	498 (6.5)	113 (8.2)	385 (6.1)
HER2-positive (non-luminal)	205 (2.7)	45 (3.3)	160 (2.5)
Triple negative	432 (5.6)	100 (7.2)	332 (5.2)
Unknown	12,396	11,867	529
Type of surgery*			
Partial mastectomy	15,079 (77.3)	9,359 (74.0)	5,720 (83.3)
Mastectomy	4,440 (22.7)	3,295 (26.0)	1,145 (16.7)
Unknown	595	594	1
Adjuvant radiotherapy*			

No	2,810 (18.3)	1,946 (21.3)	864 (14.0)
Yes	12,509 (81.7)	7,199 (78.7)	5,310 (86.0)
Unknown	4,795	4103	692
Adjuvant endocrine therapy*			
No	5,781 (46.1)	3,173 (49.6)	2,608 (42.5)
Yes	6,753 (53.9)	3,221 (50.4)	3,532 (57.5)
Unknown	7,580	6854	726
Adjuvant chemotherapy*			
No	10,709 (92.9)	5,213 (96.2)	5,496 (89.9)
Yes	824 (7.1)	204 (3.8)	620 (10.1)
Unknown	8,581	7,831	750
Adjuvant trastuzumab*			
Yes	329 (NA)	11 (NA)	318 (NA)
Unknown	19,785	13,237	6,548

*) Percentages are calculated without including women in the unknown category in the denominator.

†) Women with missing registry data on menopausal status aged <45 were considered premenopausal whereas those aged ≥55 postmenopausal.

‡) Data on whether the tumor was screening detected or clinically detected is restricted to women aged 40-74 years.

§) Luminal A = ER-positive, PR-positive, HER2-negative, grade 1-2; Luminal B (HER2-negative) = ER-positive, HER2-negative, and PR-negative and/or grade 3; Luminal B (HER2-positive) = ER-positive, HER2-positive; HER2-positive (non-luminal) = ER-negative, PR-negative and HER2-positive; triple-negative = ER-negative, PR-negative and HER2-negative.

Supplementary Table 3. Baseline characteristics among 13,248 women with T1abN0 breast cancer in the regional breast cancer registries overall and stratified by region.							
**Characteristic**	**Total No. (%)**	**Stockholm-Gotland No. (%)**	**Uppsala-Örebro No. (%)**	**South-East No. (%)**	**South No. (%)**	**West No. (%)**	**North No. (%)**
Total, No.	13,248	3,720	2,232	1,717	2,289	2,124	1,166
Year of diagnosis, min-max	1977-2007	1977-2007	1992-2007	1983-2007	1982-2007	1989-2007	1987-2007
Age at diagnosis, y							
<35	132 (1.0)	50 (1.3)	16 (0.7)	19 (1.1)	15 (0.7)	21 (1.0)	11 (0.9)
35-44	864 (6.5)	298 (8.0)	133 (6.0)	118 (6.9)	136 (5.9)	101 (4.8)	78 (6.77)
45-54	3,109 (23.5)	887 (23.8)	528 (23.7)	341 (19.9)	531 (23.2)	548 (25.8)	274 (23.5)
55-64	4,436 (33.5)	1,249 (33.6)	755 (33.8)	516 (30.1)	765 (33.4)	737 (34.7)	414 (35.5)
65-74	3,909 (29.5)	931 (25.0)	658 (29.5)	616 (35.9)	689 (30.1)	636 (29.9)	379 (32.5)
≥75	798 (6.0)	305 (8.2)	142 (6.4)	107 (6.2)	153 (6.7)	81 (3.8)	10 (0.9)
Menopausal status*†							
Premenopausal	1,981 (16.9)	835 (23.4)	426 (20.2)	137 (10.0)	149 (8.0)	345 (18.0)	89 (10.0)
Postmenopausal	9,732 (83.1)	2,727 (76.6)	1,680 (79.8)	1,239 (90.0)	1,710 (92.0)	1,573 (82.0)	803 (90.0)
Unknown	1,535	158	126	341	430	206	274
Screening detected*‡							
Yes	4,432 (78.4)	0 (NA)	1,541 (76.1)	734 (NA)	462 (74.8)	1,442 (72.8)	253 (86.6)
No	1,219 (21.6)	0 (NA)	484 (23.9)	0 (NA)	156 (25.2)	540 (27.2)	39 (13.4)
Unknown	7,597	3,720	207	983	1,671	142	874
Tumor size							
≤5 mm	2,145 (16.2)	653 (17.6)	390 (17.5)	274 (16.0)	340 (14.9)	321 (15.1)	167 (14.3)
6-10 mm	11,103 (83.8)	3,067 (82.4)	1,842 (82.5)	1,443 (84.0)	1,949 (85.1)	1,803 (84.9)	999 (85.7)
Tumor grade*							
I	2,463 (43.1)	239 (40.9)	608 (42.3)	363 (50.0)	352 (41.0)	566 (53.3)	335 (31.9)
II	2,451 (42.9)	276 (47.2)	632 (43.9)	288 (39.7)	381 (44.4)	381 (35.9)	493 (47.0)
III	804 (14.1)	70 (12.0)	198 (13.8)	75 (10.3)	125 (14.6)	115 (10.8)	221 (21.1)

Unknown	7,530	3,135	794	991	1,431	1,062	117
ER status*							
Positive	7,042 (84.9)	2,146 (84.3)	1,505 (86.0)	611 (74.9)	658 (88.3)	1,565 (88.4)	557 (83.8)
Negative	1,252 (15.1)	400 (15.7)	246 (14.0)	205 (25.1)	87 (11.7)	206 (11.6)	108 (16.2)
Unknown	4,954	1,174	481	901	1,544	353	501
PR status*							
Positive	5,475 (68.5)	1,601 (68.2)	1,208 (69.4)	478 (58.5)	542 (73.7)	1,217 (69.1)	429 (72.0)
Negative	2,522 (31.5)	745 (31.8)	533 (30.6)	339 (41.5)	193 (26.3)	545 (30.9)	167 (28.0)
Unknown	5,251	1,374	491	900	1,554	362	570
HER2 status*							
Positive	166 (11.1)	0 (NA)	61 (9.9)	41 (15.0)	11 (13.8)	30 (11.9)	23 (8.3)
Negative	1,334 (88.9)	0 (NA)	555 (90.1)	233 (85.0)	69 (86.3)	222 (88.1)	255 (91.7)
Unknown	11,748	3,720	1,616	1,443	2,209	1,872	888
Proliferation*							
Low (Ki67 ≤20%)	632 (73.6)	0 (NA)	390 (74.6)	0 (NA)	0 (NA)	0 (NA)	242 (72.0)
High (Ki67 >20%)	227 (26.4)	0 (NA)	133 (25.4)	0 (NA)	0 (NA)	0 (NA)	94 (28.0)
Unknown	12,389	3,720	1,709	1,717	2,289	2,124	830
Intrinsic subgroups*§							
Luminal A	843 (61.0)	0 (NA)	370 (62.0)	120 (50.8)	49 (63.6)	152 (63.6)	152 (63.6)
Luminal B (HER2-negative)	280 (20.3)	0 (NA)	129 (21.6)	54 (22.9)	10 (13.0)	41 (17.2)	46 (19.2)
Luminal B (HER2-positive)	113 (8.2)	0 (NA)	48 (8.0)	26 (11.0)	4 (5.2)	24 (10.0)	11 (4.6)
HER2-positive (non-luminal)	45 (3.3)	0 (NA)	13 (2.2)	10 (4.2)	7 (9.1)	6 (2.5)	9 (3.8)
Triple negative	100 (7.2)	0 (NA)	37 (6.2)	26 (11.0)	7 (9.1)	16 (6.7)	14 (5.9)
Unknown	11,867	3,720	1,635	1,481	2,212	1,885	934
Type of surgery*							
Partial mastectomy	9,359 (74.0)	2,538 (68.3)	1,825 (81.8)	678 (59.7)	1,708 (74.6)	1,696 (80.0)	914 (78.5)
Mastectomy	3,295 (26.0)	1,176 (31.7)	407 (18.2)	457 (40.3)	581 (25.4)	423 (20.0)	251 (21.5)
Unknown	594	6	0	582	0	5	1

Adjuvant radiotherapy*							
No	1,946 (21.3)	832 (27.2)	0 (NA)	0 (NA)	9 (1.5)	735 (37.8)	370 (32.6)
Yes	7,199 (78.7)	2,231 (72.8)	1,628 (NA)	773 (NA)	593 (98.5)	1,209 (62.2)	765 (67.4)
Unknown	4103	657	604	944	1,687	180	31
Adjuvant endocrine therapy*							
No	3,173 (49.6)	919 (30.0)	0 (NA)	0 (NA)	27 (36.5)	1246 (77.8)	981 (87.5)
Yes	3,221 (50.4)	2,142 (70.0)	424 (NA)	113 (NA)	47 (63.5)	355 (22.2)	140 (12.5)
Unknown	6854	659	1,808	1,604	2,215	523	45
Adjuvant chemotherapy*							
No	5,213 (96.2)	2,673 (98.2)	0 (NA)	0 (NA)	28 (80.0)	1,419 (96.7)	1,093 (97.4)
Yes	204 (3.8)	50 (1.8)	61 (NA)	9 (NA)	7 (20.0)	48 (3.3)	29 (2.6)
Unknown	7,831	997	2,171	1,708	2,254	657	44
Adjuvant trastuzumab*							
Yes	11 (NA)	0 (NA)	9 (NA)	0 (NA)	0 (NA)	1 (NA)	1 (NA)
Unknown	13,237	3,720	2,223	1,717	2,289	2,123	1,165

*) Percentages are calculated without including women in the unknown category in the denominator.

†) Women with missing registry data on menopausal status aged <45 were considered premenopausal and those aged ≥55 as postmenopausal.

‡) Data on whether the tumor was screening detected or clinically detected is restricted to women aged 40-74 years.

§) Luminal A = ER-positive, PR-positive, HER2-negative, grade 1-2; Luminal B (HER2-negative) = ER-positive, HER2-negative, and PR-negative and/or grade 3; Luminal B (HER2-positive) = ER-positive, HER2-positive; HER2-positive (non-luminal) = ER-negative, PR-negative and HER2-positive; triple-negative = ER-negative, PR-negative and HER2-negative.

Supplementary Table 4. Adjuvant treatment by selected tumor characteristics among 20,114 women with T1abN0 breast cancer.												
	**Type of surgery**			**Radiotherapy**			**Endocrine treatment**			**Chemotherapy**		
**Characteristic**	Partial mastectomy	Mastect-omy	Un-known	No	Yes	Un-known	No	Yes	Un-known	No	Yes	Un-known
Tumor size												
≤5 mm	2,267 (69.8)	980 (30.2)	120	537 (22.1)	1,895 (77.9)	935	1,057 (53.3)	926 (46.7)	1,384	1,729 (93.4)	122 (6.6)	1,516
6-10 mm	12,812 (78.7)	3,460 (21.3)	475	2,273 (17.6)	10,614 (82.4)	3,860	4,724 (44.8)	5,827 (55.2)	6,196	8,980 (92.7)	702 (7.3)	7,065
Tumor grade												
I	4,491 (86.4)	708 (13.6)	217	520 (11.9)	3,837 (88.1)	1,059	1,922 (52.0)	1,777 (48.0)	1,717	3,438 (98.9)	39 (1.1)	1,939
II	4,307 (82.0)	944 (18.0)	160	644 (14.4)	3,829 (85.6)	938	1,457 (36.5)	2,537 (63.5)	1,417	3,412 (93.1)	252 (6.9)	1,747
III	1,184 (74.6)	403 (25.4)	44	271 (20.3)	1,067 (79.7)	293	612 (51.8)	570 (48.2)	449	671 (59.0)	467 (41.0)	493
Unknown	5,097 (68.1)	2,385 (31.9)	174	1,375 (26.7)	3,776 (73.3)	2,505	1,790 (49.1)	1,869 (50.9)	3,997	3,188 (98.0)	66 (2.0)	4,402
ER-status												
Positive	10,512 (80.6)	2,526 (19.4)	217	1,865 (17.2)	8,969 (82.8)	2,421	3,743 (39.1)	5,830 (60.9)	3,682	8,363 (95.3)	409 (4.7)	4,483
Negative	1,210 (68.9)	545 (31.1)	49	265 (19.7)	1,078 (80.3)	461	901 (87.8)	125 (12.2)	778	640 (62.7)	381 (37.3)	783
Unknown	3,357 (71.0)	1,369 (29.0)	329	680 (21.6)	2,462 (78.4)	1,913	1,137 (58.8)	798 (41.2)	3,120	1,706 (98.0)	34 (2.0)	3,315
HER2-status												
Positive	466 (67.4)	225 (32.6)	30	140 (23.7)	450 (76.3)	131	196 (34.9)	365 (65.1)	160	162 (30.8)	364 (69.2)	195
Negative	5,979 (84.9)	1,064 (15.1)	166	730 (11.6)	5,545 (88.4)	934	2,426 (41.3)	3,447 (58.7)	1,336	5,266 (94.5)	304 (5.5)	1,639

Unknown	8,634 (73.3)	3,151 (26.7)	399	1,940 (22.9)	6,514 (77.1)	3,730	3,159 (51.8)	2,941 (48.2)	6,084	5,281 (97.1)	156 (2.9)	6,747
Intrinsic subgroups*												
Luminal A	4,500 (86.4)	707 (13.6)	93	531 (11.4)	4,138 (88.6)	631	1,765 (40.1)	2,635 (59.9)	900	4,137 (98.8)	51 (1.2)	1,112
Luminal B (HER2- negative)	1,022 (82.8)	213 (17.2)	48	136 (12.3)	970 (87.7)	177	312 (29.7)	737 (70.3)	234	881 (91.6)	81 (8.4)	321
Luminal B (HER2-positive)	345 (72.5)	131 (27.5)	22	87 (20.4)	339 (79.6)	72	52 (12.7)	357 (87.3)	89	131 (35.7)	236 (64.3)	131
HER2-positive (non-luminal)	111 (55.8)	88 (44.2)	6	50 (33.1)	101 (66.9)	54	135 (96.4)	5 (3.6)	65	27 (18.5)	119 (81.5)	59
Triple negative	322 (77.2)	95 (22.8)	15	39 (11.0)	314 (89.0)	79	282 (94.3)	17 (5.7)	133	143 (46.3)	166 (53.7)	123
Unknown	8,779 (73.2)	3,206 (26.8)	411	1,967 (22.8)	6,647 (77.2)	3,782	3,235 (51.9)	3,002 (48.1)	6,159	5,390 (96.9)	171 (3.1)	6,835

*) Luminal A = ER-positive, PR-positive, HER2-negative, grade 1-2; Luminal B (HER2-negative) = ER-positive, HER2-negative, and PR-negative and/or grade 3; Luminal B (HER2-positive) = ER-positive, HER2-positive; HER2-positive (non-luminal) = ER-negative, PR-negative and HER2-positive; triple-negative = ER-negative, PR-negative and HER2-negative.

**Supplementary Table 5. Adjuvant treatment by selected tumor characteristics among 6,866 women with T1abN0 breast cancer in the National Breast Cancer Registry.**												
	**Type of surgery**			**Radiotherapy**			**Endocrine treatment**			**Chemotherapy**		
**Characteristic**	Partial mastectomy	Mastect-omy	Un-known	No	Yes	Un-known	No	Yes	Un-known	No	Yes	Un-known
Tumor size												
≤5 mm	893 (73.1)	329 (26.9)	0	161 (16.1)	838 (83.9)	223	530 (53.4)	463 (46.6)	229	903 (91.0)	89 (9.0)	230
6-10 mm	4,827 (85.5)	816 (14.5)	1	703 (13.6)	4,472 (86.4)	469	2,078 (40.4)	3,069 (59.6)	497	4,593 (89.6)	531 (10.4)	520
Tumor grade												
I	2,582 (87.4)	371 (12.6)	0	294 (11.2)	2,329 (88.8)	330	1,335 (51.2)	1,273 (48.8)	345	2,574 (98.8)	31 (1.2)	348
II	2,472 (83.5)	487 (16.5)	1	385 (14.2)	2,331 (85.8)	244	867 (32.1)	1,835 (67.9)	258	2,479 (92.1)	213 (7.9)	268
III	588 (71.1)	239 (28.9)	0	167 (22.3)	581 (77.7)	79	364 (49.0)	379 (51.0)	84	365 (49.7)	369 (50.3)	93
Unknown	78 (61.9)	48 (38.1)	0	18 (20.7)	69 (79.3)	39	42 (48.3)	45 (51.7)	39	78 (91.8)	7 (8.2)	41
ER-status												
Positive	5,266 (84.8)	946 (15.2)	1	770 (13.7)	4,871 (86.3)	572	2,116 (37.7)	3,497 (62.3)	600	5,247 (93.8)	345 (6.2)	621
Negative	390 (70.7)	162 (29.3)	0	88 (18.9)	378 (81.1)	86	438 (95.0)	23 (5.0)	91	184 (40.2)	274 (59.8)	94
Unknown	64 (63.4)	37 (36.6)	0	6 (9.0)	61 (91.0)	34	54 (81.8)	12 (18.2)	35	65 (98.5)	1 (1.5)	35
HER2-status												
Positive	375 (67.6)	180 (32.4)	0	134 (27.1)	361 (72.9)	60	184 (37.6)	306 (62.4)	65	148 (30.6)	336 (69.4)	71
Negative	5,023 (85.5)	851 (14.5)	1	683 (12.8)	4,664 (87.2)	528	2,220 (41.7)	3,099 (58.3)	556	5,024 (94.8)	273 (5.2)	578

Unknown	322 (73.9)	114 (26.1)	0	47 (14.2)	285 (85.8)	104	204 (61.6)	127 (38.4)	105	324 (96.7)	11 (3.3)	101
Intrinsic subgroups*												
Luminal A	3,869 (86.8)	587 (13.2)	1	504 (12.4)	3,560 (87.6)	393	1,631 (40.3)	2,417 (59.7)	409	3,991 (98.8)	47 (1.2)	419
Luminal B (HER2- negative)	834 (83.2)	169 (16.8)	0	128 (13.8)	797 (86.2)	78	286 (31.2)	632 (68.8)	85	837 (92.1)	72 (7.9)	94
Luminal B (HER2-positive)	281 (73.0)	104 (27.0)	0	84 (23.7)	271 (76.3)	30	50 (14.3)	300 (85.7)	35	123 (35.8)	221 (64.2)	41
HER2-positive (non-luminal)	86 (53.8)	74 (46.3)	0	49 (37.7)	81 (62.3)	30	126 (96.9)	4 (3.1)	30	22 (16.9)	108 (83.1)	30
Triple negative	261 (78.6)	71 (21.4)	0	36 (12.4)	254 (87.6)	42	270 (94.7)	15 (5.3)	47	134 (47.3)	149 (52.7)	49
Unknown	389 (73.5)	140 (26.5)	0	63 (15.4)	347 (84.6)	119	245 (59.9)	164 (40.1)	120	389 (94.4)	23 (5.6)	117

*) Luminal A = ER-positive, PR-positive, HER2-negative, grade 1-2; Luminal B (HER2-negative) = ER-positive, HER2-negative, and PR-negative and/or grade 3; Luminal B (HER2-positive) = ER-positive, HER2-positive; HER2-positive (non-luminal) = ER-negative, PR-negative and HER2-positive; triple-negative = ER-negative, PR-negative and HER2-negative.

Supplementary Table 6. Cumulative incidences (%) and hazard ratios of death from breast cancer by patient and tumor characteristics among 20,112 women with T1abN0 breast cancer when women diagnosed with a metachronous breast cancer were censored at the time of diagnosis of the metachronous breast cancer (sensitivity analysis 1).					
	**Cumulative incidence, % (95% confidence interval)**			**Hazard ratio (95% confidence interval)**	
**Characteristic**	**10 years**	**20 years**	**30 years**	**Simple model***	**Full model†**
All	3.1 (2.8-3.4)	6.3 (5.8-6.8)	8.4 (7.6-9.2)	NA	NA
Age at diagnosis, y, events/N				746/20,112	168/11,188
<35	9.9 (5.5-15.8)	24.7 (15.7-34.7)	-	3.36 (2.20-5.12)	1.36 (0.42-4.44)
35-44	4.5 (3.3-6.0)	9.8 (7.6-12.3)	-	1.50 (1.15-1.97)	1.32 (0.73-2.39)
45-54	3.1 (2.6-3.8)	5.9 (4.9-6.9)	-	ref.	ref.
55-64	2.7 (2.3-3.2)	6.2 (5.4-7.1)	-	1.10 (0.90-1.35)	0.99 (0.66-1.48)
65-74	2.8 (2.3-3.3)	5.6 (4.8-6.4)	6.2 (5.3-7.3)	1.16 (0.94-1.43)	0.98 (0.64-1.52)
≥75	4.8 (3.5-6.3)	-	-	2.18 (1.58-3.01)	1.63 (0.78-3.39)
Menopausal status, events/N				673/18,134	148/10,126
Premenopausal	3.8 (3.0-4.7)	8.8 (7.4-10.4)	-	ref.	ref.
Postmenopausal	3.0 (2.7-3.3)	5.9 (5.4-6.5)	7.2 (6.4-8.0)	1.04 (0.71-1.50)	1.64 (0.78-3.49)
Screening detected, events/N				269/11,948	108/8,813
No	3.0 (2.2-3.9)	6.6 (4.9-8.5)	-	ref.	ref.
Yes	2.5 (2.1-2.9)	5.5 (4.7-6.4)	-	0.88 (0.66-1.18)	1.23 (0.76-1.98)
Tumor size, events/N				746/20,112	168/11,188
≤5 mm	3.3 (2.6-4.1)	6.0 (4.9-7.2)	-	ref.	ref.
6-≤10 mm	3.1 (2.8-3.4)	6.4 (5.9-7.0)	8.7 (7.8-9.7)	1.05 (0.86-1.28)	0.77 (0.52-1.14)
Tumor grade, events/N				244/12,456	168/11,188
I	1.2 (0.8-1.7)	4.8 (2.3-8.6)	-	ref.	ref.
II	3.1 (2.4-3.8)	7.0 (5.1-9.3)	-	2.33 (1.64-3.30)	2.57 (1.68-3.93)
III	6.7 (5.3-8.5)	13.8 (10.3-17.9)	-	4.95 (3.43-7.16)	4.64 (2.86-7.52)
ER-status, events/N				431/15,057	168/11,188
Negative	6.3 (5.1-7.6)	9.4 (7.7-11.2)	-	ref.	ref.
Positive	2.3 (2.0-2.7)	5.8 (5.1-6.6)	8.7 (7.4-10.2)	0.58 (0.47-0.72)	0.66 (0.45-0.97)
PR-status, events/N				397/14,755	160/11,051
Negative	4.8 (4.1-5.7)	8.6 (7.3-10.0)	11.4 (9.4-13.6)	ref.	ref.

Positive	1.8 (1.5-2.2)	4.9 (4.1-5.7)	-	0.48 (0.39-0.59)	0.41 (0.29-0.60)
HER2-status, events/N‡	5 years	NA	NA	43/7,477	42/7,332
Negative	0.6 (0.4-0.8)	-	-	ref.	ref.
Positive	2.8 (1.4-4.7)	-	-	3.84 (1.95-7.55)	1.94 (0.93-4.04)
Intrinsic subgroups, events/N‡	5 years	NA	NA	41/7,332	41/7,286
Luminal A	0.2 (0.1-0.5)	-	-	ref.	ref.
Luminal B (HER2-negative)	1.3 (0.6-2.4)	-	-	4.20 (1.78-9.94)	3.42 (1.36-8.58)
Luminal B (HER2-positive)	2.1 (0.9-4.4)	-	-	7.37 (2.79-19.5)	5.32 (1.93-14.7)
HER2-positive (non-luminal)	NA	-	-	12.2 (4.13-35.9)	7.14 (2.15-23.7)
Triple negative	3.4 (1.6-6.5)	-	-	9.41 (3.70-24.0)	5.95 (2.11-16.8)

*) The simple model is adjusted for year of diagnosis (continuous) age at diagnosis (categorical: <35, 35-44, 45-54, 55-64, 65-74, ≥75), region (categorical: Stockholm-Gotland, Uppsala-Örebro, South-East, South, West, North) and registry source (categorical: regional breast cancer registry, the National Breast Cancer Registry).

†) The full model is adjusted for the same variables as the simple model plus tumor size (categorical: T1a, t1b), tumor grade (categorical: I, II, III) and ER-status (categorical: positive, negative).

‡) Analyses of HER2-status and the intrinsic subgroups is restricted to women diagnosed January 1, 2005 onwards.

- Data not available for analysis

Supplementary Table 7. Cumulative incidences (%) and hazard ratios of death from breast cancer by patient and tumor characteristics among 18,998 women with T1abN0 breast cancer; excluding women with any prior cancer, except non-melanoma skin cancer and cancer in situ of the cervix, at study entry (sensitivity analysis 2).					
	**Cumulative incidence, % (95% confidence interval)**			**Hazard ratio (95% confidence interval)**	
**Characteristic**	**10 years**	**20 years**	**30 years**	**Simple model***	**Full model†**
All	3.4 (3.0-3.6)	7.6 (7.0-8.1)	10.6 (9.7-11.5)	NA	NA
Age at diagnosis, y, events/N				862/18,998	168/10,493
<35	11.2 (6.5-17.2)	26.7 (17.9-36.3)	-	3.14 (2.12-4.65)	1.67 (0.59-4.70)
35-44	5.0 (3.7-6.6)	11.0 (8.7-13.5)	18.0 (13.6-22.9)	1.44 (1.12-1.85)	1.28 (0.71-2.32)
45-54	3.1 (2.6-3.8)	7.0 (6.0-8.1)	-	ref.	ref.
55-64	2.9 (2.5-3.4)	7.6 (6.6-8.6)	10.5 (8.9-12.2)	1.16 (0.96-1.39)	0.98 (0.65-1.46)
65-74	3.1 (2.6-3.7)	7.1 (6.2-8.0)	8.1 (6.9-9.3)	1.25 (1.03-1.52)	1.12 (0.73-1.71)
≥75	4.8 (3.4-6.5)	-	-	1.90 (1.36-2.65)	1.53 (0.68-3.45)
Menopausal status, events/N				772/17,100	147/9,488
Premenopausal	4.1 (3.3-5.1)	10.0 (8.5-11.6)	16.4 (13.4-19.6)	ref.	ref.
Postmenopausal	3.2 (2.8-3.5)	7.1 (6.5-7.8)	9.1 (8.2-10.0)	0.92 (0.65-1.31)	1.79 (0.83-3.88)
Screening detected, events/N				303/11,268	106/8,275
No	2.8 (2.0-3.8)	8.1 (6.2-10.2)	-	ref.	ref.
Yes	2.7 (2.3-3.2)	6.6 (5.7-7.6)	-	0.89 (0.68-1.17)	1.21 (0.74-1.97)
Tumor size, events/N				862/18,998	168/11,493
≤5 mm	3.4 (2.7-4.2)	6.8 (5.6-8.1)	-	ref.	ref.
6-≤10 mm	3.3 (3.0-3.7)	7.7 (7.1-8.3)	10.9 (9.9-12.0)	1.09 (0.91-1.32)	0.70 (0.47-1.03)
Tumor grade, events/N				248/11,697	168/10,493
I	1.1 (0.8-1.7)	4.9 (2.5-8.4)	-	ref.	ref.
II	3.1 (2.4-3.8)	7.8 (5.8-10.1)	-	2.34 (1.66-3.30)	2.67 (1.74-4.10)
III	7.2 (5.6-9.0)	13.8 (10.4-17.8)	-	4.78 (3.31-6.90)	5.01 (3.08-8.15)
ER-status, events/N				482/14,175	168/10,493
Negative	6.8 (5.5-8.2)	10.3 (8.5-12.3)	-	ref.	ref.
Positive	2.4 (2.1-2.8)	7.1 (6.3-8.0)	10.9 (9.4-12.6)	0.67 (0.55-0.83)	0.71 (0.49-1.05)
PR-status, events/N				46/13,887	159/10,363
Negative	5.1 (4.3-6.0)	9.5 (8.2-11.0)	12.5 (10.5-14.8)	ref.	ref.

Positive	2.0 (1.6-2.4)	6.3 (5.4-7.3)	-	0.57 (0.47-0.70)	0.48 (0.33-0.70)
HER2-status, events/N‡	5 years	NA	NA	39/6,976	38/6,846
Negative	0.5 (0.3-0.8)	-	-	ref.	ref.
Positive	2.9 (1.5-5.0)	-	-	4.61 (2.34-9.07)	2.37 (1.13-4.98)
Intrinsic subgroups, events/N‡	5 years	NA	NA	37/6,842	37/6,800
Luminal A	0.2 (0.1-0.5)	-	-	ref.	ref.
Luminal B (HER2-negative)	0.8 (0.3-1.7)	-	-	2.61 (0.99-6.90)	1.92 (0.68-5.46)
Luminal B (HER2-positive)	2.2 (0.9-4.6)	-	-	7.70 (3.01-19.7)	5.19 (1.92-14.1)
HER2-positive (non-luminal)	-	-	-	11.6 (3.93-34.2)	5.86 (1.71-20.1)
Triple negative	-	-	-	8.19 (3.11-21.6)	4.69 (1.57-14.0)

*) The simple model is adjusted for year of diagnosis (continuous) age at diagnosis (categorical: <35, 35-44, 45-54, 55-64, 65-74, ≥75), region (categorical: Stockholm-Gotland, Uppsala-Örebro, South-East, South, West, North) and registry source (categorical: regional breast cancer registry, the National Breast Cancer Registry).

†) The full model is adjusted for the same variables as the simple model plus tumor size (categorical: T1a, t1b), tumor grade (categorical: I, II, III) and ER-status (categorical: positive, negative).

‡) Analyses of HER2-status and the intrinsic subgroups is restricted to women diagnosed January 1, 2005 onwards.

- Data not available for analysis

Supplementary Table 8. Cumulative incidences (%) and hazard ratios of death from breast cancer by patient and tumor characteristics among 12,085 women with T1abN0 breast cancer diagnosed January 1, 2000 onwards (sensitivity analysis 3).					
	**Cumulative incidence, % (95% confidence interval)**			**Hazard ratio (95% confidence interval)**	
**Characteristic**	**10 years**	**20 years**	**30 years**	**Simple model***	**Full model†**
All	2.2 (1.9-2.6)	-	-	NA	NA
Age at diagnosis, y, events/N				166/12,085	121/10,498
<35	-	-	-	3.74 (1.32-10.6)	1.19 (0.28-5.10)
35-44	3.6 (1.8-6.4)	-	-	1.92 (1.03-3.58)	1.55 (0.75-3.18)
45-54	1.9 (1.2-2.8)	-	-	ref.	ref.
55-64	2.1 (1.6-2.8)	-	-	1.12 (0.72-1.74)	1.11 (0.67-1.85)
65-74	2.2 (1.5-3.0)	-	-	1.23 (0.78-1.95)	1.15 (0.67-1.97)
≥75	-	-	-	2.14 (1.09-4.20)	1.85 (0.82-4.16)
Menopausal status, events/N				153/10,945	111/9,556
Premenopausal	2.6 (1.6-4.0)	-	-	ref.	ref.
Postmenopausal	2.3 (1.9-2.8)	-	-	1.64 (0.72-3.73)	1.68 (0.66-4.30)
Screening detected, events/N				97/9,059	81/8,380
No	2.0 (1.2-3.3)	-	-	ref.	ref.
Yes	2.0 (1.5-2.6)	-	-	1.16 (0.68-1.96)	1.23 (0.70-2.16)
Tumor size, events/N				166/12,085	121/10,498
≤5 mm	2.7 (1.9-3.8)	-	-	ref.	ref.
6-≤10 mm	2.1 (1.7-2.5)	-	-	0.77 (0.53-1.12)	0.75 (0.48-1.18)
Tumor grade, events/N				139/11,109	121/10,498
I	0.9 (0.6-1.5)	-	-	ref.	ref.
II	2.5 (1.9-3.2)	-	-	2.75 (1.73-4.37)	2.51 (1.53-4.13)
III	6.1 (4.3-8.2)	-	-	7.73 (4.74-12.6)	5.24 (2.91-9.45)
ER-status, events/N				134/11,189	121/10,498
Negative	5.9 (4.1-8.0)	-	-	ref.	ref.
Positive	1.7 (1.3-2.1)	-	-	0.26 (0.18-0.38)	0.47 (0.29-0.74)
PR-status, events/N				135/11,098	121/10,409
Negative	4.3 (3.4-5.5)	-	-	ref.	ref.
Positive	1.3 (1.0-1.8)	-	-	0.23 (0.16-0.33)	0.34 (0.22-0.52)

HER2-status, events/N‡	5 years	NA	NA	45/7,477	44/7,332
Negative	0.6 (0.4-0.8)	-	-	ref.	ref.
Positive	2.7 (1.4-4.7)	-	-	3.87 (2.01-7.45)	2.01 (0.99-4.07)
Intrinsic subgroups, events/N‡	5 years	NA	NA	43/7,332	43/7,286
Luminal A	0.2 (0.1-0.5)	-	-	ref.	ref.
Luminal B (HER2-negative)	1.3 (0.6-2.4)	-	-	3.71 (1.60-8.60)	2.87 (1.16-7.09)
Luminal B (HER2-positive)	2.1 (0.9-4.3)	-	-	7.27 (2.90-18.2)	5.03 (1.92-13.2)
HER2-positive (non-luminal)	-	-	-	10.7 (3.68-31.0)	5.76 (1.76-18.9)
Triple negative	3.4 (1.5-6.4)	-	-	8.20 (3.28-20.5)	4.89 (1.76-13.6)

*) The simple model is adjusted for year of diagnosis (continuous) age at diagnosis (categorical: <35, 35-44, 45-54, 55-64, 65-74, ≥75), region (categorical: Stockholm-Gotland, Uppsala-Örebro, South-East, South, West, North) and registry source (categorical: regional breast cancer registry, the National Breast Cancer Registry).

†) The full model is adjusted for the same variables as the simple model plus tumor size (categorical: T1a, t1b), tumor grade (categorical: I, II, III) and ER-status (categorical: positive, negative).

‡) Analyses of HER2-status and the intrinsic subgroups is restricted to women diagnosed January 1, 2005 onwards.

- Data not available for analysis

Supplementary Table 9. Cumulative incidences (%) and hazard ratios of death from breast cancer by patient and tumor characteristics among 6,864 women with T1abN0 breast cancer in the National Breast Cancer Registry (sensitivity analysis 4).					
	**Cumulative incidence, % (95% confidence interval)**			**Hazard ratio (95% confidence interval)**	
**Characteristic**	**5 years**	**NA**	**NA**	**Simple model***	**Full model†**
All	0.7 (0.5-1.0)	-	-	NA	NA
Age at diagnosis, y, events/N				30/6,864	29/6,664
<35	-	-	-	NA	NA
35-44	-	-	-	1.17 (0.31-4.42)	0.91 (0.24-3.46)
45-54	1.1 (0.4-2.5)	-	-	ref.	ref.
55-64	0.5 (0.2-1.1)	-	-	0.39 (0.14-1.12)	0.44 (0.15-1.26)
65-74	0.6 (0.3-1.2)	-	-	0.64 (0.25-1.63)	0.81 (0.31-2.09)
≥75	-	-	-	1.02 (0.27-3.86)	0.81 (0.17-3.86)
Menopausal status, events/N				28/6,421	27/6,240
Premenopausal	1.4 (0.6-2.9)	-	-	ref.	ref.)
Postmenopausal	0.6 (0.4-1.0)	-	-	0.80 (0.18-3.60)	0.79 (0.17-3.77)
Screening detected, events/N				26/6,297	26/6,121
No	0.9 (0.4-1.8)	-	-	ref.	ref.
Yes	0.6 (0.3-1.0)	-	-	0.90 (0.36-2.26)	1.06 (0.42-2.67)
Tumor size, events/N				30/6,864	29/6,664
≤5 mm	-	-	-	ref.	ref.
6-≤10 mm	0.7 (0.4-1.1)	-	-	0.91 (0.37-2.22)	0.83 (0.33-2.06)
Tumor grade, events/N				29/6,738	29/6,664
I	-	-	-	ref.	ref.
II	0.8 (0.4-1.4)	-	-	4.43 (1.26-15.6)	4.15 (1.18-14.6)
III	2.9 (1.5-5.0)	-	-	16.5 (4.66-58.6)	9.70 (2.39-39.3)
ER-status, events/N				30/6,763	29/6,664
Negative	4.2 (2.2-7.2)	-	-	ref.	ref.
Positive	0.5 (0.3-0.8)	-	-	0.13 (0.06-0.28)	0.35 (0.14-0.88)
PR-status, events/N				30/6,758	29/6,654
Negative	2.3 (1.4-3.6)	-	-	ref.	ref.
Positive	0.4 (0.2-0.9)	-	-	0.16 (0.08-0.34)	0.29 (0.11-0.75)

HER2-status, events/N				28/6,428	28/6,333
Negative	0.5 (0.3-0.8)	-	-	ref.	ref.
Positive	-	-	-	3.98 (1.73-9.13)	1.71 (0.70-4.15)
Intrinsic subgroups, events/N				27/6,335	27/6,304
Luminal A	0.4 (0.1-1.1)	-	-	ref.	ref.
Luminal B (HER2-negative)	1.4 (0.6-3.0)	-	-	5.73 (1.98-16.6)	4.46 (1.40-14.2)
Luminal B (HER2-positive)	-	-	-	7.27 (2.04-25.9)	4.87 (1.29-18.4)
HER2-positive (non-luminal)	-	-	-	16.4 (4.55-59.0)	8.88 (2.08-37.9)
Triple negative	2.9 (1.0-6.8)	-	-	11.0 (3.33-36.4)	6.29 (1.59-24.8)

*) The simple model is adjusted for year of diagnosis (continuous) age at diagnosis (categorical: <35, 35-44, 45-54, 55-64, 65-74, ≥75), region (categorical: Stockholm-Gotland, Uppsala-Örebro, South-East, South, West, North) and registry source (categorical: regional breast cancer registry, the National Breast Cancer Registry).

†) The full model is adjusted for the same variables as the simple model plus tumor size (categorical: T1a, t1b), tumor grade (categorical: I, II, III) and ER-status (categorical: positive, negative).

- Data not available for analysis

**Supplementary Table 10. Cumulative incidences (%) and hazard ratios, calculated using the Fine and Gray approach to account for competing events, of death from breast cancer by patient and tumor characteristics among 20,112 women with T1abN0 breast cancer (sensitivity analysis 5).**					
	**Cumulative incidence, % (95% confidence interval)**			**Hazard ratio (95% confidence interval)**	
**Characteristic**	**10 years**	**20 years**	**30 years**	**Simple model***	**Full model†**
All	3.4 (3.1-3.7)	7.6 (7.1-8.2)	10.5 (9.6-11.4)	NA	NA
Year of diagnosis, events/N				915/20,112	190/11,188
1977-1989	5.5 (4.6-6.5)	10.6 (9.3-11.9)	13.6 (12.2-15.2)	ref.	ref.
1990-1994	3.9 (3.3-4.7)	8.3 (7.4-9.3)	-	0.68 (0.57-0.81)	0.54 (0.20-1.48)
1995-1999	4.0 (3.3-4.8)	-	-	0.59 (0.48-0.72)	0.43 (0.17-1.07)
2000-2004	2.5 (1.9-3.1)	-	-	0.39 (0.30-0.49)	0.24 (0.09-0.59)
2005-	-	-	-	0.30 (0.21-0.42)	0.18 (0.07-0.48)
Age at diagnosis, y, events/N				915/20,112	190/11,188
<35	11.2 (6.5-17.2)	26.7 (17.9-36.3)	-	3.30 (2.24-4.87)	1.57 (0.60-4.09)
35-44	5.3 (3.9-6.9)	11.6 (9.3-14.2)	18.6 (14.1-23.5)	1.56 (1.23-2.00)	1.42 (0.82-2.45)
45-54	3.2 (2.7-3.9)	7.1 (6.1-8.2)	-	ref.	ref.
55-64	3.0 (2.5-3.5)	7.7 (6.7-8.7)	10.4 (8.9-12.1)	1.06 (0.88-1.26)	0.91 (0.62-1.34)
65-74	3.1 (2.6-3.6)	7.0 (6.1-7.9)	7.9 (6.9-9.1)	0.93 (0.78-1.13)	0.93 (0.63-1.39)
≥75	5.1 (3.8-6.7)	-	-	1.05 (0.78-1.43)	1.20 (0.60-2.40)
Menopausal status, events/N				822/18,134	166/10,126
Premenopausal	4.3 (3.5-5.2)	10.5 (9.0-12.1)	16.7 (13.8-19.9)	ref.	ref.
Postmenopausal	3.2 (2.9-3.6)	7.2 (6.6-7.8)	9.0 (8.1-9.9)	0.86 (0.60-1.22)	1.53 (0.73-3.20)
Screening detected, events/N				327/11,948	121/8,813
No	3.0 (2.2-4.0)	8.1 (6.3-10.1)	-	ref.	ref.
Yes	2.7 (2.3-3.2)	6.9 (5.9-7.9)	-	0.89 (0.68-1.15)	1.20 (0.75-1.91)
Tumor size, events/N				915/20,112	190/11,188
≤5 mm	3.6 (2.9-4.4)	6.9 (5.7-8.2)	-	ref.	ref.
6-≤10 mm	3.4 (3.0-3.7)	7.8 (7.2-8.4)	10.8 (9.9-11.9)	1.07 (0.89-1.28)	0.72 (0.49-1.05)
Tumor grade, events/N				275/12,456	190/11,188
I	1.2 (0.9-1.8)	5.2 (2.9-8.5)	-	ref.	ref.
II	3.2 (2.6-3.9)	7.9 (6.0-10.2)	-	2.21 (1.60-3.05)	2.43 (1.65-3.58)

III	7.4 (5.9-9.2)	14.0 (10.6-17.8)	-	4.45 (3.13-6.33)	4.28 (2.74-6.68)
ER-status, events/N				516/15,057	190/11,188
Negative	7.0 (5.7-8.4)	10.5 (8.8-12.5)	-	ref.	ref.
Positive	2.5 (2.2-2.9)	7.2 (6.4-8.0)	10.8 (9.3-12.4)	0.65 (0.52-0.80)	0.69 (0.47-1.00)
PR-status, events/N				478/14,755	181/11,051
Negative	5.3 (4.5-6.2)	9.8 (8.5-11.2)	12.7 (10.7-14.8)	ref.	ref.
Positive	2.0 (1.7-2.4)	6.3 (5.5-7.3)	-	0.55 (0.45-0.66)	0.44 (0.31-0.62)
HER2-status, events/N‡	5 years	NA	NA	45/7,477	44/7,332
Negative	0.6 (0.4-0.8)	-	-	ref.	ref.
Positive	2.7 (1.4-4.7)	-	-	3.86 (2.07-7.19)	2.01 (0.90-4.47)
Intrinsic subgroups, events/N‡	5 years	NA	NA	43/7,332	43/7,286
Luminal A	0.2 (0.1-0.5)	-	-	ref.	ref.
Luminal B (HER2-negative)	1.3 (0.6-2.4)	-	-	3.72 (1.58-8.77)	2.89 (1.10-7.59)
Luminal B (HER2-positive)	2.1 (0.9-4.3)	-	-	7.29 (3.05-17.4)	5.07 (1.85-13.9)
HER2-positive (non-luminal)	-	-	-	10.5 (3.63-30.5)	5.78 (1.72-19.4)
Triple negative	3.4 (1.5-6.4)	-	-	8.20 (3.24-20.7)	4.93 (1.88-12.9)

*) The simple model is adjusted for year of diagnosis (continuous) age at diagnosis (categorical: <35, 35-44, 45-54, 55-64, 65-74, ≥75), region (categorical: Stockholm-Gotland, Uppsala-Örebro, South-East, South, West, North) and registry source (categorical: regional breast cancer registry, the National Breast Cancer Registry).

†) The full model is adjusted for the same variables as the simple model plus tumor size (categorical: T1a, t1b), tumor grade (categorical: I, II, III) and ER-status (categorical: positive, negative).

‡) Analyses of HER2-status and the intrinsic subgroups is restricted to women diagnosed January 1, 2005 onwards.

- Data not available for analysis

Supplementary Table 11. Cumulative incidences (%) and hazard ratios of death from breast cancer by patient and tumor characteristics among 9,264 women with T1abN0 breast cancer, starting follow-up at 10 years after diagnosis among women having survived and not being censored at that time.

Characteristic	Cumulative incidence, % (95% confidence interval)			Hazard ratio (95% confidence interval)	
	**10 years**	**20 years**	**30 years**	**Simple model***	**Full model†**
All	5.0 (4.5-5.6)	8.5 (7.5-9.5)	-	NA	NA
Year of diagnosis, events/N				422/9,264	43/2,214
1977-1989	6.5 (5.4-7.7)	10.3 (8.9-11.9)	-	ref.	ref.
1990-1994	5.2 (4.4-6.1)	-	-	0.71 (0.55-0.91)	0.42 (0.09-1.95)
1995-1999	-	-	-	0.54 (0.40-0.74)	0.44 (0.09-2.17)
2000-2004	-	-	-	0.49 (0.30-0.79)	0.35 (0.06-1.90)
2005-	-	-	-	-	-
Age at diagnosis, y, events/N				422/9,264	43/2,214
<35	18.5 (10.3-28.6)	-	-	3.19 (1.85-5.52)	2.05 (0.26-16.4)
35-44	6.9 (4.9-9.3)	14,4 (10.0-19.6)	-	1.51 (1.07-2.13)	2.11 (0.76-5.87)
45-54	4.1 (3.3-5.2)	-	-	ref.	ref.
55-64	5.3 (4.4-6.3)	8.4 (6.8-10.2)	-	1.33 (1.02-1.72)	1.14 (0.50-2.59)
65-74	5.1 (4.2-6.2)	6.3 (5.1-7.7)	-	1.52 (1.16-2.00)	1.57 (0.68-3.64)
≥75	-	-	-	1.29 (0.60-2.78)	-
Menopausal status, events/N				380/8,139	37/1,882
Premenopausal	6.6 (5.3-8.2)	13.4 (10.4-16.7)	-	ref.	ref.
Postmenopausal	4.9 (4.3-5.5)	7.1 (6.2-8.2)	-	0.67 (0.41-1.10)	0.46 (0.11-1.98)
Screening detected, events/N				134/3,859	24/1,440
No	5.7 (4.0-7.9)	-	-	ref.	ref.
Yes	4.8 (3.9-5.9)	-	-	0.88 (0.59-1.31)	1.99 (0.68-5.80)
Tumor size, events/N				422/9,264	43/2,214
≤5 mm	3.9 (2.9-5.2)	-	-	ref.	ref.
6-≤10 mm	5.3 (4.7-5.9)	8.9 (7.8-10.1)	-	1.39 (1.04-1.87)	1.25 (0.44-3.54)
Tumor grade, events/N				75/3,105	43/2,214
I	4.6 (2.1-8.6)	-	-	ref.	ref.
II	5.6 (3.6-8.3)	-	-	1.34 (0.77-2.33)	1.88 (0.87-4.08)

III	8.5 (5.0-13.2)	-	-	1.65 (0.87-3.11)	2.60 (1.02-6.61)
ER-status, events/N				217/5,283	43/2,214
Negative	4.4 (3.0-6.3)	-	-	ref.	ref.
Positive	5.4 (4.6-6.4)	9.7 (8.1-11.6)	-	1.55 (1.06-2.27)	1.96 (0.79-4.86)
PR-status, events/N				200/5,062	39/2,123
Negative	5.5 (4.2-7.0)	9.0 (6.9-11.5)	-	ref.	ref.
Positive	5.0 (4.1-6.1)	-	-	0.96 (0.71-1.28)	0.46 (0.22-0.95)

*) The simple model is adjusted for year of diagnosis (continuous) age at diagnosis (categorical: <35, 35-44, 45-54, 55-64, 65-74, ≥75), region (categorical: Stockholm-Gotland, Uppsala-Örebro, South-East, South, West, North) and registry source (categorical: regional breast cancer registry, the National Breast Cancer Registry).

†) The full model is adjusted for the same variables as the simple model plus tumor size (categorical: T1a, t1b), tumor grade (categorical: I, II, III) and ER-status (categorical: positive, negative).

- Data not available for analysis

Supplementary Table 12. Cumulative incidences (%) and hazard ratios of death from any cause by patient and tumor characteristics among 20,114 women with T1abN0 breast cancer.

	Cumulative incidence, % (95% confidence interval)			Hazard ratio (95% confidence interval)	
**Characteristic**	**10 years**	**20 years**	**30 years**	**Simple model***	**Full model†**
All	14.4 (13.9-15.0)	40.1 (39.1-41.1)	67.4 (65.6-69.1)	NA	NA
Age at diagnosis, y, events/N				5,416/20,114	1,162/11,190
<35	11.0 (6.6-16.8)	25.2 (17.1-34.1)	-	1.00 (0.69-1.44)	0.95 (0.39-2.34)
35-44	6.3 (4.9-8.0)	15.4 (12.8-18.2)	30.5 (25.2-36.0)	0.81 (0.68-0.97)	0.81 (0.53-1.23)
45-54	6.5 (5.7-7.3)	15.8 (14.3-17.3)	37.3 (33.1-41.5)	ref.	ref.
55-64	9.9 (9.1-10.7)	33.2 (31.4-35.0)	68.5 (64.8-71.8)	2.30 (2.09-2.53)	1.82 (1.47-2.25)
65-74	20.4 (19.3-21.6)	62.5 (60.5-64.3)	95.0 (92.9-96.5)	5.39 (4.91-5.91)	3.71 (3.03-4.54)
≥75	49.0 (45.6-52.2)	95.4 (92.9-97.1)	-	15.6 (13.9-17.5)	10.9 (8.61-13.7)
Menopausal status, events/N				5,135/18,136	1,079/10,128
Premenopausal	5.9 (5.0-6.9)	15.5 (13.7-17.3)	30.3 (26.5-34.2)	ref.	ref.
Postmenopausal	17.2 (16.5-17.9)	49.0 (47.8-50.2)	80.5 (78.5-82.3)	1.50 (1.24-1.82)	1.79 (1.14-2.82)
Screening detected, events/N				1,894/11,949	691/8,814
No	10.2 (8.8-11.8)	30.3 (27.1-33.5)	-	ref.	ref.
Yes	12.1 (11.3-13.0)	38.5 (36.6-40.4)	-	0.99 (0.88-1.11)	1.09 (0.89-1.33)
Tumor size, events/N				5,416/20,114	1,162/11,190
≤5 mm	14.3 (12.9-15.7)	37.7 (35.2-40.2)	62.8 (58.5-66.8)	ref.	ref.
6-≤10 mm	14.4 (13.8-15.1)	40.5 (39.4-41.6)	68.5 (66.4-70.4)	1.01 (0.94-1.09)	1.01 (0.85-1.19)
Tumor grade, events/N				1,556/12,458	1,162/11,190
I	10.8 (9.7-11.9)	33.1 (29.0-37.2)	-	ref.	ref.
II	13.4 (12.3-14.7)	38.0 (34.2-41.8)	-	1.20 (1.07-1.34)	1.18 (1.04-1.34)
III	17.0 (14.8-19.4)	40.7 (35.7-45.6)	-	1.60 (1.38-1.85)	1.56 (1.30-1.88)
ER-status, events/N				3,079/15,059	1,162/11,190
Negative	17.6 (15.7-19.6)	39.4 (36.2-42.5)	62.0 (56.7-66.8)	ref.	ref.
Positive	13.0 (12.3-13.7)	41.0 (39.3-42.6)	69.0 (66.1-71.8)	0.90 (0.81-0.99)	0.88 (0.74-1.06)
PR-status, events/N				2,901/14,757	1,129/11,053
Negative	16.1 (14.8-17.5)	42.8 (40.3-45.3)	70.3 (66.5-73.8)	ref.	ref.
Positive	12.3 (11.6-13.2)	39.1 (37.3-40.9)	65.3 (61.2-69.1)	0.92 (0.85-1.00)	0.93 (0.80-1.08)

HER2-status, events/N‡	5 years	10 years	NA	410/7,479	397/7,334
Negative	4.3 (3.8-4.9)	11.1 (9.7-12.5)	-	ref.	ref.
Positive	5.1 (3.4-7.2)	-	-	1.14 (0.81-1.59)	1.00 (0.70-1.43)
Intrinsic subgroups, events/N‡	5 years	10 years	NA	398/7,334	393/7,288
Luminal A	4.0 (3.4-4.7)	10.4 (8.9-12-1)	-	ref.	ref.
Luminal B (HER2-negative)	4.6 (3.4-6.1)	10.8 (8.2-13.8)	-	1.09 (0.84-1.42)	0.93 (0.69-1.26)
Luminal B (HER2-positive)	4.7 (2.8-7.3)	-	-	1.08 (0.71-1.65)	0.96 (0.62-1.48)
HER2-positive (non-luminal)	7.4 (3.8-12.5)	-	-	1.50 (0.88-2.58)	1.11 (0.61-2.05)
Triple negative	6.5 (4.1-9.5)	15.9 (8.5-25.3)	-	1.38 (0.94-2.02)	1.08 (0.69-1.68)

*) The simple model is adjusted for year of diagnosis (continuous) age at diagnosis (categorical: <35, 35-44, 45-54, 55-64, 65-74, ≥75), region (categorical: Stockholm-Gotland, Uppsala-Örebro, South-East, South, West, North) and registry source (categorical: regional breast cancer registry, the National Breast Cancer Registry).

†) The full model is adjusted for the same variables as the simple model plus tumor size (categorical: T1a, t1b), tumor grade (categorical: I, II, III) and ER-status (categorical: positive, negative).

‡) Analyses of HER2-status and the intrinsic subgroups is restricted to women diagnosed January 1, 2005 onwards.

- Data not available for analysis

**Supplementary Table 13. Cumulative incidences (%) and hazard ratios of metachronous breast cancer by patient and tumor characteristics among 20,112 women with T1abN0 breast cancer.**					
	**Cumulative incidence, % (95% confidence interval)**			**Hazard ratio (95% confidence interval)**	
**Characteristic**	**10 years**	**20 years**	**30 years**	**Simple model***	**Full model†**
All	5.1 (4.8-5.5)	10.2 (9.6-10.8)	12.8 (11.9-13.7)	NA	NA
Age at diagnosis, y, events/N				1,236/20,112	371/11,188
<35	3.8 (1.4-8.1)	16.8 (9.7-25.7)	-	1.30 (0.79-2.15)	0.68 (0.17-2.78)
35-44	5.8 (4.4-7.5)	11.7 (9.4-14.2)	19.2 (15.0-23.8)	1.20 (0.96-1.50)	0.94 (0.57-1.54)
45-54	5.0 (4.3-5.8)	10.9 (9.7-12.3)	15.5 (13.0-18.1)	ref.	ref.
55-64	5.2 (4.6-5.8)	11.0 (9.9-12.2)	13.6 (12.0-15.3)	1.06 (0.91-1.22)	1.04 (0.79-1.37)
65-74	5.5 (4.8-6.2)	9.3 (8.3-10.3)	-	1.10 (0.94-1.28)	1.19 (0.90-1.58)
≥75	3.7 (2.6-5.1)	-	-	0.91 (0.65-1.27)	0.96 (0.54-1.70)
Menopausal status, events/N				1,092/18,134	326/10,126
Premenopausal	5.3 (4.4-6.3)	11.8 (10.2-13.5)	17.7 (14.9-20.7)	ref.	ref.
Postmenopausal	5.1 (4.7-5.6)	9.8 (9.1-10.5)	11.3 (10.4-12.2)	0.92 (0.69-1.21)	0.83 (0.48-1.45)
Screening detected, events/N				548/11,948	253/8,813
No	4.8 (3.8-5.9)	10.7 (8.7-12.9)	-	ref.	ref.
Yes	5.4 (4.8-6.0)	10.7 (9.6-11.9)	-	1.02 (0.83-1.26)	1.07 (0.78-1.48)
Tumor size, events/N				1,236/20,112	371/11,188
≤5 mm	5.2 (4.4-6.2)	10.2 (8.8-11.8)	-	ref.	ref.
6-≤10 mm	5.1 (4.7-5.5)	10.2 (9.5-10.8)	12.5 (11.6-13.5)	0.96 (0.83-1.12)	0.85 (0.65-1.13)
Tumor grade, events/N				462/12,456	371/11,188
I	5.7 (4.9-6.6)	-	-	ref.	ref.
II	5.4 (4.6-6.3)	-	-	0.95 (0.78-1.16)	0.92 (0.74-1.14)
III	4.6 (3.3-6.1)	7.2 (5.0-10.1)	-	0.78 (0.57-1.05)	0.71 (0.49-1.04)
ER-status, events/N				721/15,057	371/11,188
Negative	5.8 (4.7-7.2)	8.6 (7.0-10.4)	-	ref.	ref.
Positive	4.9 (4.4-5.4)	10.5 (9.6-11.5)	13.0 (11.6-14.5)	1.04 (0.84-1.28)	0.83 (0.59-1.18)
PR-status, events/N				702/14,755	362/11,051
Negative	5.4 (4.6-6.3)	9.1 (7.8-10.5)	-	ref.	ref.
Positive	5.0 (4.4-5.5)	10.8 (9.8-12.0)	14.0 (12.0-16.2)	1.01 (0.86-1.19)	0.98 (0.75-1.29)

HER2-status, events/N‡	5 years	NA	NA	111/7,477	107/7,332
Negative	2.0 (1.6-2.5)	-	-	ref.	ref.
Positive	2.0 (0.9-3.9)	-	-	1.05 (0.56-1.96)	0.95 (0.48-1.91)
Intrinsic subgroups, events/N‡	5 years	NA	NA	108/7,332	107/7,286
Luminal A	1.8 (1.3-2.3)	-	-	ref.	ref.
Luminal B (HER2-negative)	2.8 (1.8-4.2)	-	-	1.50 (0.94-2.38)	1.47 (0.88-2.44)
Luminal B (HER2-positive)	1.7 (0.5-4.1)	-	-	1.21 (0.58-2.54)	1.25 (0.58-2.73)
HER2-positive (non-luminal)	-	-	-	0.74 (0.18-3.03)	0.73 (0.17-3.20)
Triple negative	2.6 (1.1-5.1)	-	-	1.41 (0.68-2.95)	1.26 (0.53-3.02)

*) The simple model is adjusted for year of diagnosis (continuous) age at diagnosis (categorical: <35, 35-44, 45-54, 55-64, 65-74, ≥75), region (categorical: Stockholm-Gotland, Uppsala-Örebro, South-East, South, West, North) and registry source (categorical: regional breast cancer registry, the National Breast Cancer Registry).

†) The full model is adjusted for the same variables as the simple model plus tumor size (categorical: T1a, t1b), tumor grade (categorical: I, II, III) and ER-status (categorical: positive, negative).

‡) Analyses of HER2-status and the intrinsic subgroups is restricted to women diagnosed January 1, 2005 onwards.

- Data not available for analysis
